# Diversity Outbred Mice at 21: Maintaining Allelic Variation in the Face of Selection

**DOI:** 10.1534/g3.116.035527

**Published:** 2016-09-29

**Authors:** Elissa J. Chesler, Daniel M. Gatti, Andrew P. Morgan, Marge Strobel, Laura Trepanier, Denesa Oberbeck, Shannon McWeeney, Robert Hitzemann, Martin Ferris, Rachel McMullan, Amelia Clayshultle, Timothy A. Bell, Fernando Pardo-Manuel de Villena, Gary A. Churchill

**Affiliations:** * The Jackson Laboratory, Bar Harbor, Maine 04609; † Department of Genetics, Lineberger Comprehensive Cancer Center, University of North Carolina at Chapel Hill, North Carolina 27599-7264; ‡ Department of Behavioral Neuroscience and Portland Alcohol Research Center, Oregon Health & Science University, Oregon 97201

**Keywords:** transmission ratio distortion, meiotic drive, aneuploidy, Multiparent Advanced Generation Inter-Cross (MAGIC), multiparental populations MPP

## Abstract

Multi-parent populations (MPPs) capture and maintain the genetic diversity from multiple inbred founder strains to provide a resource for high-resolution genetic mapping through the accumulation of recombination events over many generations. Breeding designs that maintain a large effective population size with randomized assignment of breeders at each generation can minimize the impact of selection, inbreeding, and genetic drift on allele frequencies. Small deviations from expected allele frequencies will have little effect on the power and precision of genetic analysis, but a major distortion could result in reduced power and loss of important functional alleles. We detected strong transmission ratio distortion in the Diversity Outbred (DO) mouse population on chromosome 2, caused by meiotic drive favoring transmission of the WSB/EiJ allele at the *R2d2* locus. The distorted region harbors thousands of polymorphisms derived from the seven non-WSB founder strains and many of these would be lost if the sweep was allowed to continue. To ensure the utility of the DO population to study genetic variation on chromosome 2, we performed an artificial selection against WSB/EiJ alleles at the *R2d2* locus. Here, we report that we have purged the WSB/EiJ allele from the drive locus while preserving WSB/EiJ alleles in the flanking regions. We observed minimal disruption to allele frequencies across the rest of the autosomal genome. However, there was a shift in haplotype frequencies of the mitochondrial genome and an increase in the rate of an unusual sex chromosome aneuploidy. The DO population has been restored to genome-wide utility for genetic analysis, but our experience underscores that vigilant monitoring of similar genetic resource populations is needed to ensure their long-term utility.

The power of genetic mapping studies in model organism populations derives, in large part, from uniform and high allele frequencies at all variant loci across the genome. Multi-parent populations (MPPs) such as the Diversity Outbred (DO) mouse population provide high mapping precision due to the accumulation of recombination events across multiple breeding generations. It is important to maintain allelic balance during the breeding process and this can be achieved by maintaining a large effective population size with randomized matings (Rockman and Kruglyak 2008). The founding generation (G0) of the DO population consisted of randomly chosen mice from the incipient Collaborative Cross (CC) breeding lines, which were derived from eight inbred founder strains (Collaborative Cross Consortium 2012; Svenson *et al.* 2012). Software-assisted breeding has facilitated adherence to the randomized breeding design of the DO. However, natural phenomena such as allelic incompatibility between loci or meiotic drive have the potential to directionally disrupt allelic balance more rapidly than expected from stochastic genetic drift, which would require hundreds of generations to substantially alter allele frequencies in this population.

## Discovery of transmission ratio distortion (TRD) in the DO

Several thousand DO mice have been genotyped across multiple studies spanning from generations G4 through G21. Data sharing among the DO user community enabled us to monitor the autosomal and sex chromosomal haplotype distributions over time.

In generations G8 and G9, we observed a substantial and growing distortion of allele frequencies on chromosome 2. An excess of WSB/EiJ alleles had been previously noted in the CC lines, where it appeared to have stabilized at a frequency of ∼0.20 (Aylor *et al.* 2011; Collaborative Cross Consortium 2012). However, the frequency of WSB/EiJ alleles in the DO continued to rise and, by G12, exceeded 0.60 (more than five times the expected value of 0.125). The cause for this rapid sweep was identified as a novel meiotic drive locus named *R2d2* (*responder to meiotic drive on chromosome 2*; Didion *et al.* 2015). *R2d2* is a copy number variant; in a permissive genetic background, alleles with high copy number (including WSB/EiJ) are subject to TRD through the maternal germline due to meiotic drive. It appeared likely that, without some intervention, the WSB/EiJ allele would sweep to fixation and as a result genetic variation in the DO would be depleted across a large portion of chromosome 2. Further support for this conclusion was provided by a similar distortion in allele frequency observed in the region spanning the *R2d2* locus in a related outbred population known as the Heterogeneous Stock-Collaborative Cross (HS-CC), which has undergone many additional generations of outbreeding (Supplemental Material, Figure S1) (Iancu *et al.* 2010).

The fixation of a single haplotype across a large genomic region in a mapping population would create a blind spot with no detectable genetic variation. To date, over 145 quantitative trait loci (QTL) have been mapped to genetic variants on chromosome 2 [(URL: http://www.informatics.jax.org), 2/25/2016] and 730 features with phenotypic alleles [URL: http://www.informatics.jax.org), 2/25/2016] are localized to this chromosome. These include protein coding genes, chromosomal deletions, miRNA genes, antisense lncRNA, chromosomal duplications, chromosomal inversions, endogenous retroviral sequences, insertions, and lincRNA genes. Fixation of a single haplotype would eliminate our ability to detect the effects of variants in this region on complex traits, and would mask the region in expression QTL and other systems used in genetic analyses. Epistatic interactions involving loci on chromosome 2 would be limited to the WSB/EiJ haplotype, thereby either masking or exaggerating their effects in the DO population.

## Remedial strategies

In order to maintain allelic variation on chromosome 2, we decided to intervene in the meiotic drive process. We sought an intervention that would deviate as little as possible from the original breeding strategy, in which two randomly selected progeny (one female and one male) from each of 175 DO lineages are assigned to new mating pairs at random with avoidance of paired siblings. Due to increasing demand for DO mice starting in generation G8, the randomized assignments were being made in duplicate such that two males and two females were drawn from each litter and assigned to mating groups “A” and “B.” Progeny from 175 mated pairs in the “A” group were selected to maintain the core DO population and progeny from mated pairs in the “B” group were used primarily for distribution. The availability of two mated pairs per lineage expanded the available pool of matings and allowed us to reduce the frequency of WSB/EiJ alleles, while avoiding a bottleneck in the core breeding population.

Several remedial strategies could be employed to mitigate the impact of meiotic drive on genetic variation. One strategy was to fix the central region of chromosome 2 with the WSB/EiJ-derived haplotype leaving the flanking regions segregating for all eight founder haplotypes. However, this strategy would leave a large “blind spot” in the middle of the chromosome due to reduced recombination in that region (Liu *et al.* 2014; Morgan *et al.* 2016b). An alternative strategy was to purge the WSB/EiJ allele, preserving genetic variation along the entire chromosome but with seven, rather than eight, founder haplotypes represented in the central region. We concluded that retaining as much segregating variation as possible was the preferred solution. We speculated that the latter strategy could be augmented by the reintroduction of a WSB/EiJ haplotype carrying a spontaneous copy number reduction at *R2d2* that would be incapable of drive (Didion *et al.* 2015).

The allelic distortion on chromosome 2 was first detected in genotypes from generations G8 and G9, but by the time these data were analyzed, the G12 matings had been established and WSB/EiJ allele frequency had exceeded 0.60 (Didion *et al.* 2016). Based on the observed rate of change, we determined that it was still possible to purge the WSB/EiJ allele at *R2d2* while maintaining the essential characteristics of the DO population—random assortment and balanced allele frequencies—across the uninvolved regions of the genome. The need to monitor the progress of the purge and its potential impact on other regions of the genome lead us to the discovery of additional irregularities in the genetic makeup of the mitochondrial genome and sex chromosomes in the DO population, which we describe below.

## Materials and Methods

### DO production colony maintenance

The DO mouse production colony is maintained at The Jackson Laboratory in a standard barrier, specific pathogen-free facility. Breeder pairs were housed in individual pens of duplex cages [pen dimensions 12″ × 6″ × 5″ (L × W × H)] on pressurized, individually ventilated racks with hardwood chip/shaving bedding. The mice were fed a Lab Diet 5K0Q (St. Louis, MO) *ad libitum* and were provided with filtered water in bottles acidified to pH 2.5–3.0. The room temperature was maintained at 70° (± 2°) and 50% humidity (± 20%), with a light cycle of 14 hr on and 10 hr off. The pups were weaned at 3 wk of age and housed in duplex cages with up to five sex-segregated animals per duplex pen. The core colony is maintained in 175 lineages. At each new generation, two progeny (one female, one male) are selected at random from each lineage and assigned to mating pairs at random with avoidance of sib-mating. These progeny are selected from first litters when possible and additional litters are used to provide mice for distribution. Due to an increase in demand at generation G8, we established a second mated pair within each lineage. Progeny from the “B” matings were primarily used for distribution. Breeding records are provided in File S1.

Many users of DO mice provided their genotype data for haplotype inference using the DOQTL software (Gatti *et al.* 2014). These samples were genotyped on one of the MUGA, MegaMUGA, or GigaMUGA (Neogen, Lincoln, NE) array platforms (Morgan *et al.* 2016a). Genotype data from DO mice are being archived at http://do.jax.org, and will also be accessible from the Mouse Phenome Database (http://mpd.jax.org). We strongly encourage users of DO mice to contact E.J.C. or G.A.C. to coordinate submission of genotyping data.

The HS-CC population was bred at Oregon Health & Science University (OSHU) in the research colony of Robert Hitzemann. The HS-CC were formed from the eight CC founder strains using a pseudorandom breeding design (Iancu *et al.* 2010). The colony is maintained as 48 families using a rotational breeding strategy, *i.e.*, a male from family one is bred to a female from family two and so on. The colony is currently at G35. The HS-CC mice are housed at the Portland Veterans Affairs Medical Center (VAMC), in a nonbarrier facility under standard conditions. At G25, 88 individuals were genotyped using the MegaMUGA (Morgan *et al.* 2016a).

### Marker-assisted purge of WSB/EiJ alleles at the R2d2 locus

To perform the breeding intervention in a cost-effective manner, all mating pairs were genotyped for the presence of WSB/EiJ alleles on chromosome 2 at three SNPs: rs27943666, rs28048346, and rs28030588. Primer sequences are in File S2. Genotyping was performed by LGC Genomics (Beverly, MA). DNA was isolated using sodium hydroxide extraction followed by neutralization with Tris HCl. The genotype analysis enabled us to categorize the genotypes of each potential breeder as WSB/EiJ (W) carrier status, or all other alleles (a).

Tracking of DO matings is matrilineal. Selected DO mice from generation *N*-1 are assigned to a mate pair with an identifier of the form GN0xxx, where *N* is the generation of the expected offspring and xxx is the lineage number of the dam. The sire is selected at random (with avoidance of siblings) and his maternal lineage is noted in the breeding records. Due to the nature of the production environment, there are some gaps and minor recording errors in the breeding records. Beginning with matings that produced the G8 generation, matings were set up in duplicate. The dam and sire of the A mating are full siblings of the dam and sire, respectively, in the B mating. Initially the A mating was designated for propagating the DO population and the B matings were used to expand production capacity. However, in the event that the A mating did not produce sufficient numbers of female and male offspring for the next generation, the B mating was available as a backup. In the event that neither the A nor the B mating produced a full set of offspring, another lineage was selected to provide either the dam, the sire, or both parents for the next generation matings. In the ideal, these replacements would not occur and drift in the population would achieve its theoretical minimum value.

In order to execute the purge of WSB/EiJ alleles at *R2d2*, we deviated from this mating scheme in two ways.

First, both the A and B mate pairs were genotyped. Four of the nine mating types (Table 1) can produce at least some mice that do not carry a W allele, including progeny of the aa × aa matings, Wa × Wa matings, Wa × aa, and aa × Wa matings. Of these, only offspring of crosses with a Wa parent need to be genotyped to identify aa progeny. Progeny of the aa × WW and WW × aa matings are all Wa and do not need to be typed. Progeny of the WW × Wa and Wa × WW crosses are either WW or Wa and can be identified by genotyping.

**Table 1:** Frequencies of the available mating types in the DO population classified by the presence (W) or absence (a) of the WSB/EiJ allele at the start of the intervention, generation G12

			Female		
			WW	Wa	aa
			0.36	0.48	0.16
Male	WW	0.36	0.1296	0.1728	0.0576
	Wa	0.48	0.1728	0.2304	0.0768
	aa	0.16	0.0576	0.0768	0.0256

Second, to populate the next generation, offspring of all but the WW × WW mating types were retained, but progeny were typed as needed and those with either one or zero W alleles were selected for the next generation. To minimize the W allele frequency in the subsequent generation, only a fraction of the Wa × WW/WW × Wa progeny were retained. By prioritizing those mated pairs that reduced or retained W alleles, and by selecting specifically those that carried the minimum number of alleles, we expected to bring the allele frequency from 62 to < 15% in one generation, and theoretically to purge the W allele completely in the next generation.

The meiotic drive effect was originally reported to result in ∼66% transmission of W alleles from Wa matings based on population estimates. Further work revealed that selection at *R2d2* occurs only through the female germline and is background dependent, with transmission ratio varying from 50 to 100% in favor of the W allele depending on the mating (Didion *et al.* 2015). Therefore, marker-assisted breeding and selection required multiple generations and progressed more slowly than our original estimates.

### Haplotype reconstruction

Two litters are consistently produced in both of the production and distribution colonies. Litter sizes and sex ratios were determined from breeding records of these colonies. Analyses are based on data separated by litter.

We used the allele calls from the MegaMUGA and GigaMUGA platforms (Morgan *et al.* 2016a) as inputs to a hidden Markov model (HMM) and performed haplotype reconstruction in each DO mouse (Gatti *et al.* 2014). Briefly, we estimated the posterior probability that each mouse was in one of 36 possible unphased diplotype states, given the allele call data. The HMM produces 36 diplotype probabilities (which sum to one) for each mouse at each marker. We estimated the frequency of each founder allele along the genome by condensing the 36 diplotype probabilities to eight founder haplotype probabilities and summing these across samples.

Haplogroup assignment for the Y chromosome and mitochondrial genome was performed using linear discriminant analysis (LDA) applied to genotypes from the MegaMUGA and GigaMUGA arrays separately. Based on data from the Mouse Diversity Array (MDA) (Yang *et al.* 2011), we determined that we could discriminate between six Y-chromosome haplogroups (“A” = A/J; “BCE” = C57BL/6J, 129S1/SvImJ, NZO/HlLtJ; “D” = NOD/ShiLtJ; “F” = CAST/EiJ; “G” = PWK/PhJ; and “H” = WSB/EiJ) and five mitochondrial haplogroups (“ABCD” = A/J, C57BL/6J, 129S1/SvImJ, NOD/ShiLtJ; “E” = NZO/HlLtJ; “F” = CAST/EiJ; “G” = PWK/PhJ; and “H” = WSB/EiJ) in the DO. For the Y chromosome, we trained a classifier on data from 86 (MegaMUGA) or 66 (GigaMUGA) inbred and F1 males with known Y chromosomes. We retained only genotypes at a set of seven high-confidence probes (JAX00725028, JAX00725070, JAX00725071, JAX00725036, JAX00725040, JAX00725087, and JAX00725083) carried over from the MDA and shared on both platforms. The classifier was applied to all male DO samples jointly using the predict.lda() function from the R package MASS (Venables and Ripley 2002). For the mitochondrial genome, we used a similar procedure, using the same samples for training but retaining all 46 probes (MegaMUGA) or 32 probes (GigaMUGA) mapping to the mitochondrial genome. Haplogroup assignment routines are implemented in the *R* package “argyle” (Morgan 2016).

### Screen for R2d2 mutants and estimation of transmission ratio

The *R2d2* copy number was estimated in 71 DO G16 females carrying the WSB/EiJ allele at *R2d2* in heterozygosity using two copy number assays (Life Technologies, Carlsbad, CA, catalog numbers Mm00644079_cn and Mm00053048_cn). These assays target the *Cwc22* gene that is present at the *R2d2* locus (Didion *et al.* 2015). The female with the smallest copy number (DO-G16-107) was backcrossed for three generations to C57BL/6NJ males, selecting at each generation for heterozygous *R2d2* females. TRD was tested in the progeny of nine females from this pedigree (the original DO G16, one F_1_, three N_2_, and four N_3_) by crossing them to C57BL/6NJ males and genotyping 18–60 pups. DNA for PCR-based genotyping was performed on crude whole genomic DNA extracted by heating tissue in 100 μl of 25 mM NaOH/0.2 mM EDTA at 95° for 60 min followed by the addition of 100 μl of 40 mM Tris-HCl. The samples were then spun at 2000 rpm for 10 min and the supernatant collected for use as PCR template. The following primers were used in this study: Chr2-TRD-85.65-F, 5’-AGC CTA TAG GAT TGT GTT CTA ACC-3’, position in GRCm37 85657874 and Chr2-TRD-85.65-R, 5’-ACA GCC ATG TAC TAA TCT AAA CCT-3’, position in build GRCm37 85658306. PCR reactions contained 1.5–2 mM MgCl_2_, 0.2–0.25 mM dNTPs, 0.2–1.8 μM of each primer, and 0.5–1 units of GoTaq polymerase (Promega) in a final volume of 10–50 μl. Cycling conditions were 95°, 2 min; 35 cycles at 95, 55 and 72° for 30 sec each; with a final extension at 72°, 7 min. PCR products were loaded into a 2% agarose gel and run at 200 V for 40 min. Genotypes were scored, recorded, and we used a chi-square test to detect significant deviations from Mendelian expectations favoring the WSB/EiJ allele.

### Sex chromosome abnormalities

DO mice were classified as males or females based on the mean hybridization intensity at markers located on the X and Y chromosomes; females have low signal from Y-linked probes and higher intensity at X-linked probes than males. XO females were identified by the lack of a Y chromosome, significantly reduced overall signal intensity for markers on the entire X chromosome, and by the complete lack of heterozygosity on chromosome X. Males with partial duplication of the distal X chromosome were identified by the presence of a Y chromosome and the presence of heterozygous calls on the distal portion of the X chromosome. To rule out the possibility that heterozygous calls on the X chromosome in males were technical artifacts, we used only markers with robust performance in females. We also confirmed that males with putative duplications have female-like hybridization intensity at heterozygous markers on the distal X, consistent with the presence of two X chromosomes.

### Genome annotation

All genome coordinates are on GRCm38. SNPs in the eight DO founder strains were obtained from the Sanger Mouse Genomes Project release REL-1505 (version 5) files at ftp://ftp-mouse.sanger.ac.uk/REL-1505-SNPs_Indels/mgp.v5.merged.snps_all.dbSNP142.vcf.gz (Keane *et al.* 2011).

### Data availability

Breeding records are provided in File S1. A complete sample list is provided in File S5. Genotype data are available at http://churchill-lab.jax.org/website/Chesler_2016_DO.

## Results

### Discovery and remediation of the chromosome 2 selfish sweep

**Figure 1:**
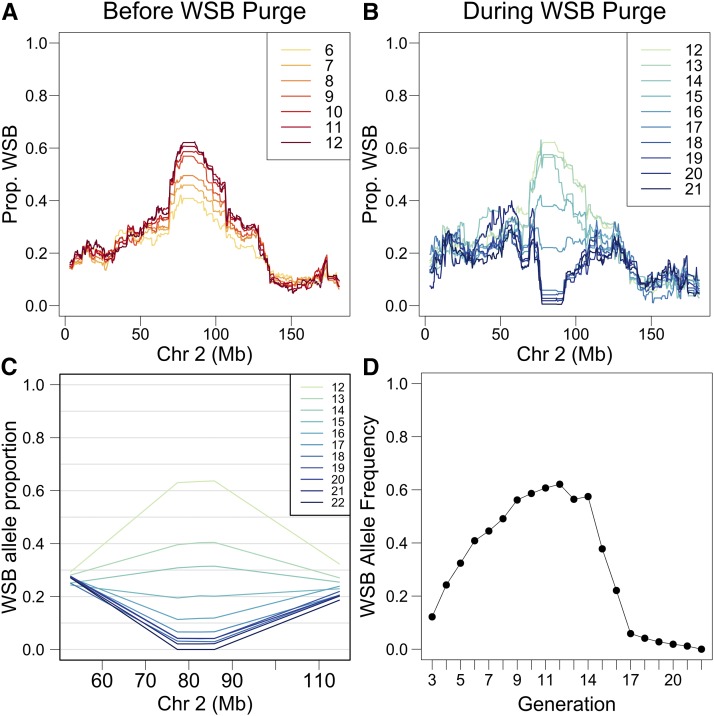
Progression of the selective sweep and purge of the WSB allele at the *R2d2* locus. (A) The WSB allele frequency at the *R2d2* locus in distributed DO mice increased through generation 12. (B) In generations 13 through 21, the WSB/EiJ allele frequency in distributed DO mice decreased as a result of intervention in the breeding design. (C) The WSB/EiJ allele frequency at selection markers flanking the *R2d2* locus shows a steady decline among breeders from generations 12–22. (D) WSB/EiJ allele frequency at the *R2d2* locus in distributed DO mice is show in profile across all generations with genotype information. Colors changing to darker shades indicate forward progression through time. Chr, chromosome; DO, Diversity Outbred.

Monitoring of DO mouse genotypes from multiple experiments revealed an increasing frequency of WSB/EiJ alleles in a region of chromosome 2 centered at ∼90 Mb (Figure 1A). Although the effect was first noticed in data from generations G8 and G9, the G12 matings were already in place and the WSB/EiJ allele frequency had reached 60% before we could begin intervention. Animals that were distributed from the DO colony through G19 retained a high but steadily decreasing frequency of WSB/EiJ alleles (Figure 1B). Marker-assisted selection of progeny was used to establish matings in the core DO colony beginning at generation G13, and WSB/EiJ allele frequency declined rapidly for the next five generations (Figure 1, C and D). By G21, WSB/EiJ allele frequencies among breeder pairs dropped to zero and the WSB/EiJ allele had been completely purged from the DO at generation G22.

The finding of a G16 DO mouse with a spontaneous mutation on the WSB/EiJ haplotype that reduced copy number at *R2d2* raised the possibility of reintroducing the WSB/EiJ haplotype to the selected region on chromosome 2. However, after comprehensive testing of maternal transmission ratio at *R2d2* in a pedigree segregating for the low copy-number allele, we concluded that the allele was still able to sustain meiotic drive (overall 159 progeny inherited the WSB/EiJ allele while 108 inherit the non-WSB/EiJ allele, *P* = 0.0018). Therefore, we suspended the plan to reintroduce the WSB/EiJ haplotype with the mutated *R2d2* allele.

### Effect of selection on the genetic structure of the DO

**Figure 2:**
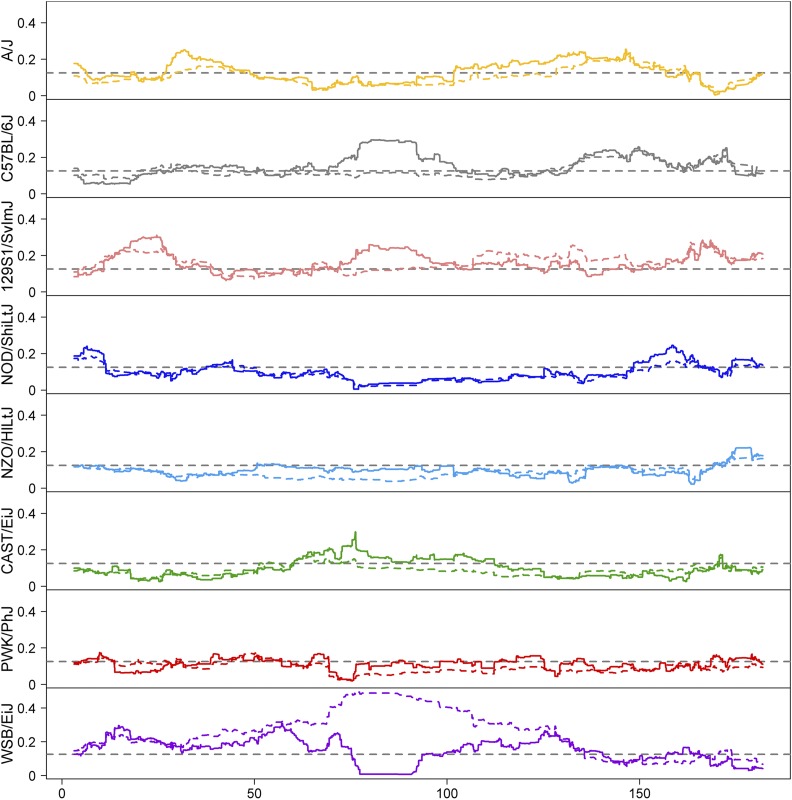
Founder haplotype frequencies across chromosome 2. Each panel shows the founder haplotype frequency at generations G11 (dashed color) and G21 (solid color) for one of the eight founder strains. The dashed gray line indicates the expected allele frequency of 0.125.

In order to understand the impact of selection against the WSB/EiJ haplotype in the region spanning *R2d2*, we examined the frequency of all eight founder haplotypes across the genome, paying particular attention to chromosome 2 (Figure 2). We chose to contrast haplotype frequencies between distributed animals from generation G21 (which at the time of writing included genome-wide profiles of 504 mice) and generation G11 mice (one generation before the start of the purge for which we have access to genome-wide profiling for 879 mice). In the targeted region of chromosome 2, the decrease in WSB/EiJ allele frequency was offset by increases in most of the remaining founder haplotypes. The highest frequencies are associated with the 129S1/SvImJ (24.4%) and C57BL/6J (29.6%) haplotypes. The wild-derived haplotypes increased from 9.8 to 13.9% for CAST and 5.8–14.9% for PWK. There is an excess of the WSB haplotype throughout most of the nonselected portion of chromosome 2 due to linkage with *R2d2*, with a peak of 31.9% proximal to *R2d2*. The remaining seven founder haplotypes are not markedly changed and there were no regions with complete loss of any founder haplotype outside of the *R2d2* region. We expect that these imbalances will persist in the DO colony with some allowance for genetic drift.

Genome-wide haplotype frequencies did not change substantially from generation G11 to G21 (File S3). The largest observed change in allele frequencies outside of chromosome 2 was an increase in the NOD haplotype from 13.6 to 33.5% over a region spanning from 90 to 100 Mb on chromosome 15.

**Figure 3:**
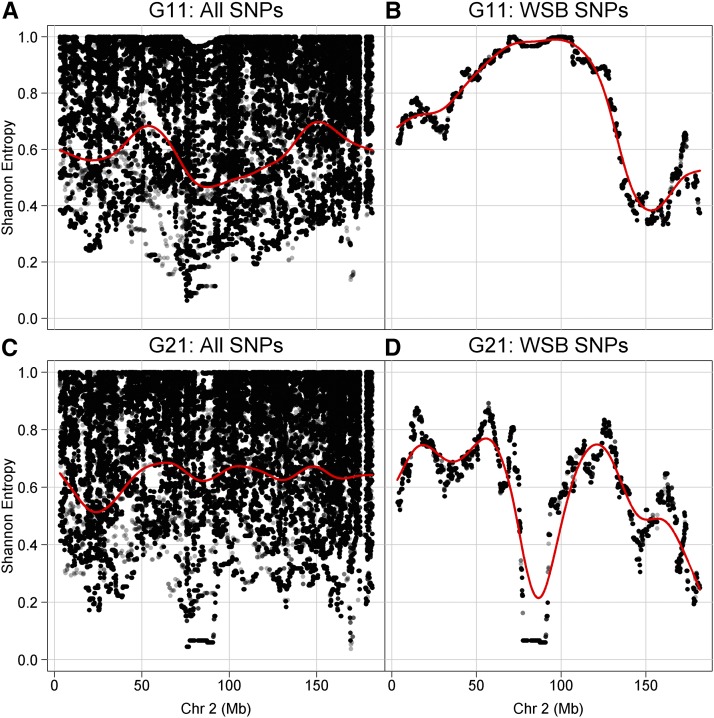
Information content of biallelic SNPs on chromosome 2. The Shannon entropy (log_2_) of individual SNPs on chromosome 2 is plotted by chromosomal position. The maximum value (1.0) is achieved for SNPs with a minor allele frequency of 1/2. Shannon entropy is plotted for all SNPs at generations G11 (A) and G21 (C), and for SNPs that are private to WSB (B and D). A smoothing spline (red) indicates average variation in information content across chromosome 2. Chr, chromosome; SNP, single nucleotide polymorphism.

The eight founder strains of the DO differ substantially in their relative contribution of unique sequence variants, with the largest contribution of variants coming from CAST/EiJ and PWK/PhJ, followed by WSB/EiJ. Thus, haplotype frequencies alone give an incomplete picture of the changes in standing genetic variation following the purge of the WSB/EiJ haplotype (Figure 3). The central region targeted by the marker-assisted purge contains 2772 SNP and 884 small indel variants that are private to WSB/EiJ. The flanking regions contain an additional 74,265 and 22,618 WSB private SNPs and indels, respectively. These alleles are lost or nearly lost in the current DO population. The distribution of minor allele frequencies for the majority of SNPs on chromosome 2 not private to WSB/EiJ remained stable throughout the selection process (Figure 3 and File S4).

### Chromosome Y and mitochondria

**Figure 4:**
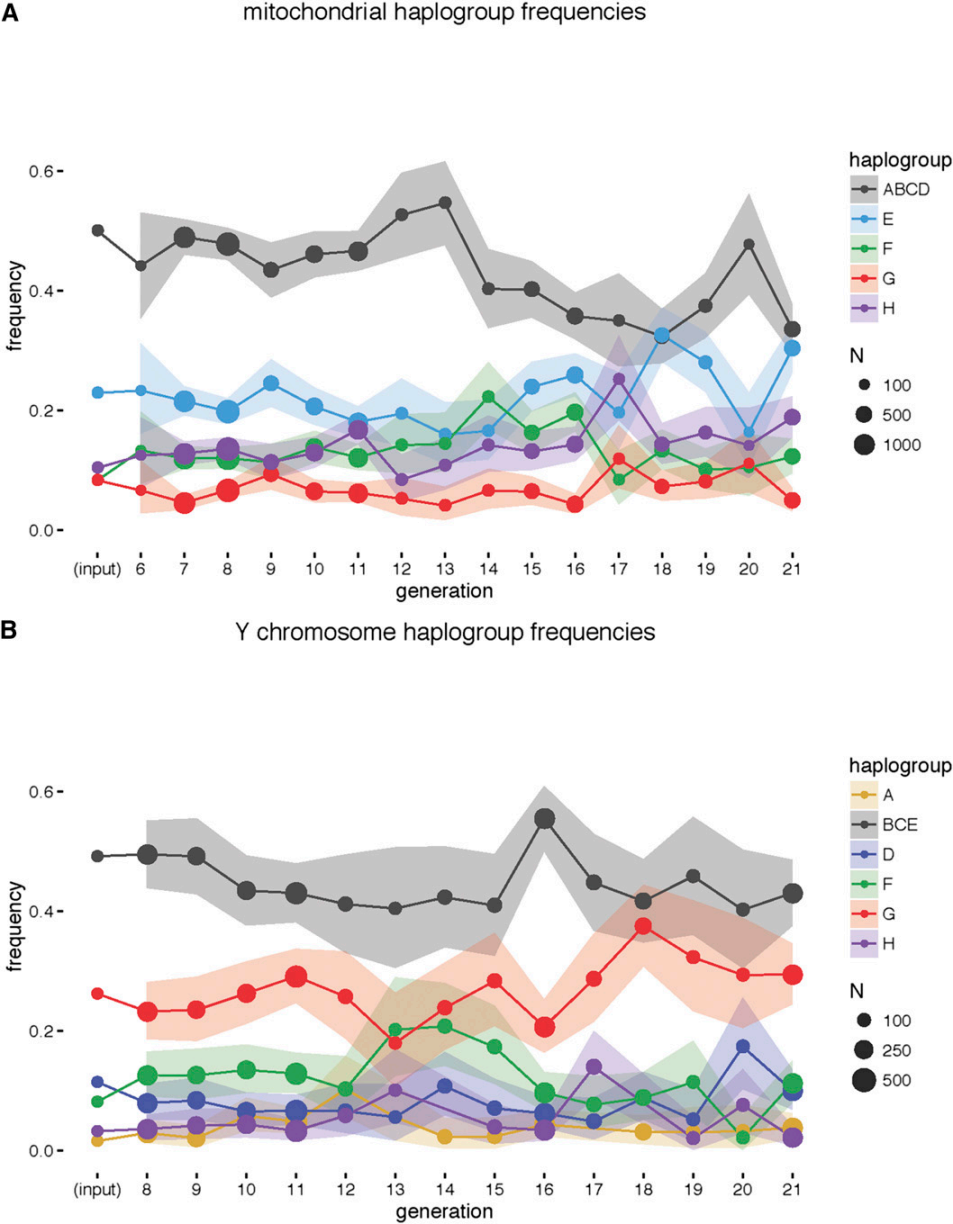
Frequencies of mitochondrial (A) and Y-chromosome (B) haplogroups by generation. Size of points indicates the number of mice sampled per generation; ribbons indicate 95% binomial C.I. for the estimated frequencies. Strains A/J, C57BL/6J, 129S1/SvImJ, and NOD/ShiLtJ (“ABCD”) share the same mitochondrial haplogroup; strains C57BL/6J, 129S1/SvImJ, and NZO/HlLtJ (“BCE”) have Y-chromosome haplogroups indistinguishable with current genotyping arrays. Input frequencies are based on the breeding structure of the CC lines used to establish the DO population. DO, Diversity Outbred.

To determine whether there are significant changes in allele frequency in the mitochondria and the Y chromosome, and to determine whether putative changes are related to the *R2d2* purge, we compared the frequencies for G6 through G21. The initial description of the DO population (Svenson *et al.* 2012) did not report the haplotype frequencies in the mitochondria and the Y chromosome due to the lack of markers in the genotyping platform used at the time (MUGA). This shortcoming was addressed in newer genotyping arrays (MegaMUGA and GigaMUGA) (Morgan *et al.* 2016a). In addition to pre- and postpurge generations, we also calculated the input frequencies based on the genotypes of incipient CC mice used to establish the DO population (Chesler *et al.* 2008; Collaborative Cross Consortium 2012; Svenson *et al.* 2012). There is a reduction in the mitochondrial haplogroup ABCD, corresponding to founder strains A/J, C57BL/6J, 129S1/SvImJ, and NOD/ShiLtJ, concomitant with the purge and strongest between G13 and G14 (Figure 4A). For the Y chromosome, there is no evidence of directional changes either before or after the purge (Figure 4B). We conclude that, despite the changes in mitochondrial haplotype frequency, the DO retains every type of Y chromosome and mitochondria. In fact, at G21, the frequencies of the four genetically distinct mitochondrial haplotypes are more evenly distributed than they were in the founding generations.

### X chromosome abnormalities

**Figure 5:**
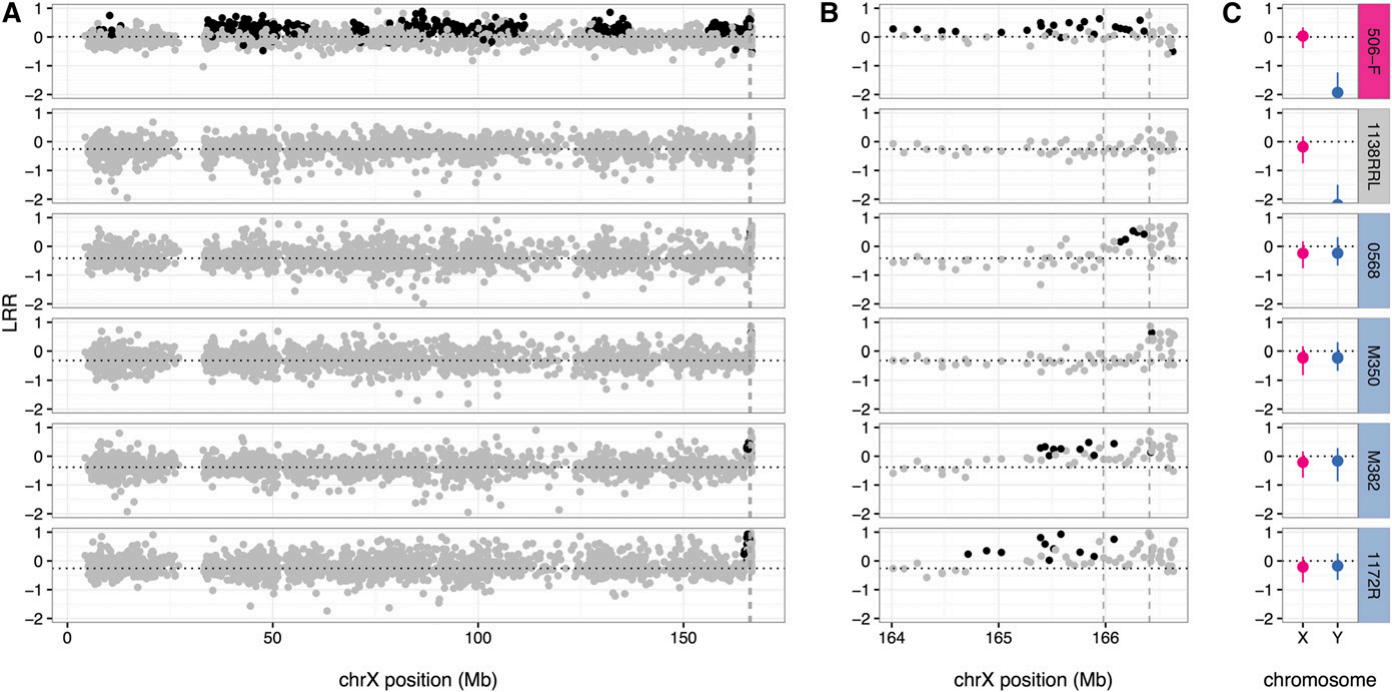
Sex-chromosome abnormalities in the DO. (A) LRR on chromosome X for (top to bottom): an XX female, an XO female, and four XY males. Black points indicate heterozygous genotype calls and gray points homozygous calls. Dotted horizontal line indicates mean LRR for each sample excluding the PAR, whose boundary is indicated by the dashed vertical lines. (B) Zoomed-in views of the distal end of chromosome X. Dashed vertical lines indicate the position of the PAR boundary in CAST/EiJ (left) and all other strains (right). Males with duplicated distal regions of the X chromosome, in the bottom two rows, have heterozygous genotypes and elevated hybridization intensity outside of the PAR. (C) Distribution of normalized hybridization intensity on chromosomes X (pink) and Y (blue) clearly differentiates karyotypes XX, XO, and XY. Panels are labeled with sample names and colored by sex-chromosome assignment: pink, XX; gray, XO; and blue, XY. chr, chromosome; DO, Diversity Outbred; LRR, Log-scale normalized hybridization intensity; PAR, pseudoautosomal region.

Examination of the genotypes on the sex chromosomes at G11 and G21 led us to the unexpected finding of DO mice with two types of sex chromosome abnormalities (Figure 5 and Table 2). Seven XO females (out of 688 total females) are characterized by the absence of heterozygosity and reduced average hybridization intensity of the SNP probes on the X chromosome. In addition, we identified 65 DO males (out of 695 total males) with apparent duplication of the distal region of the X chromosome. The rate of XO females remains constant but there is a significant increase from 4.4% in generation G11 to 15% in G21 (*P* < 0.0001) in the frequency of males with duplications of the distal X chromosome. We cannot exclude the possibility that some of these duplications may represent translocations from the X to the Y chromosome. The Y chromosome of the CAST/EiJ strain already has an expanded pseudoautosomal region (PAR) due to an X-to-Y translocation (White *et al.* 2012). Duplications of the distal X are challenging to identify in the DO due to the presence of this extended PAR in CAST/EiJ (see male 0568 in Figure 5) and the fact that the length of the duplicated region appears to vary (compare males M382 and 1172R in Figure 5).

**Table 2:** Number of DO mice with sex chromosome abnormalities (percent shown in parenthesis)

Generation	# Females	# Males	XO	X Duplication
G11	487	388	4 (0.82)	17 (4.4)
G21	194	307	3 (1.5)	48 (15.5)

### Litter size, sex ratio, and mating success in the DO

**Figure 6:**
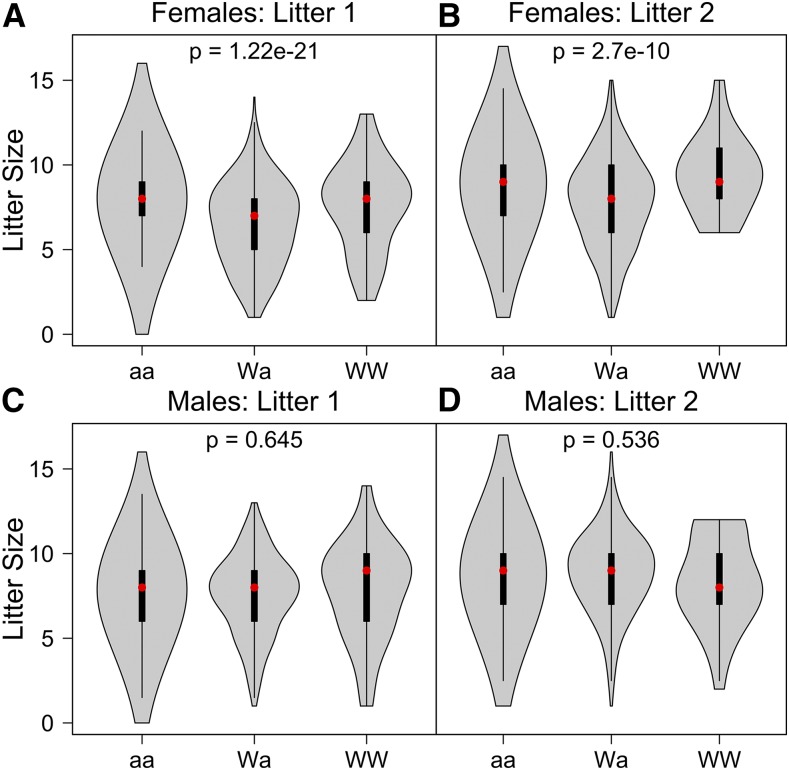
Litter size distribution among the DO breeders by allele at the *R2d2* locus. Wa alleles in dams leads to smaller litter sizes, but not in sires. The three alleles are ‘aa’ for homozygous non-WSB/EiJ, “Wa” for heterozygous WSB/EiJ, and “WW” for homozygous WSB/EiJ. Red dots are the median of each group and black boxes are the interquartile range. *P*-values are from a one-way ANOVA of litter size *vs.* genotype. ANOVA, analysis of variance; DO, Diversity Outbred.

Analysis of breeding records for more than 5000 litters across 15 generations of DO breeders (File S1) show little directional change in litter size and sex ratio. However, an effect on litter size becomes apparent when matings are partitioned according to sex and *R2d2* genotype. As expected, litter size was not found to depend on sire genotype (Litter 1: *P* = 0.645 and Litter 2: *P* = 0.536). In contrast, there is a highly significant reduction in the litter size for *R2d2* heterozygous females (Figure 6). The effect is present in both first (*P* = 1.22 × 10^−21^) and second (*P* = 2.7 × 10^−10^) litters. This result is consistent with previous reports that the level of meiotic drive observed in heterozygous females is positively correlated with a reduction in the average litter size. We conclude that fixation of the non-WSB/EiJ allele has mitigated a potentially deleterious effect of heterozygosity at the *R2d2* locus on DO maintenance and production.

The proportion of failed matings among the DO breeders varied from 3 to 7% prior and subsequent to the active intervention. However, higher rates of failure, reaching > 10% in G14, were observed during the peak of the selection process. Failed matings result in a disruption of the ideal randomized mating scheme. They reduce the effective population size and represent the only route for selection to affect mitochondrial or Y chromosome allele frequencies.

## Discussion

At 21 generations, the DO mouse population has proven to be a valuable resource for genetic mapping (Recla *et al.* 2014; Smallwood *et al.* 2014; Church *et al.* 2015; French *et al.* 2015; Gu *et al.* 2016) and systems genetics (Kelly *et al.* 2015; Chick *et al.* 2016). However, an ongoing selective sweep driven by the *R2d2* locus occurred in the DO breeding colony and threatened to eliminate genetic variation across a large region of chromosome 2. We were able to rescue most of the standing allelic variation in this region using a marker-assisted breeding strategy to purge the WSB/EiJ allele that was responsible for the sweep. Our strategy had minimal impact on independent assortment of unlinked loci, and has maintained allelic variation throughout the genome. The allele purge reversed the naturally occurring process even though WSB/EiJ allele frequencies were quite high at the time marker-based selection was initiated. Five generations were required to completely remove the WSB/EiJ haplotype at *R2d2* from the breeding colony. As of the current generation (G22), it appears that we have eliminated the meiotic drive allele but we will monitor this locus for several more generations to ensure that the purge was complete.

Although the decision to use a purge strategy retained much of the genetic variation on chromosome 2, loss of variation was inevitable. In addition to the complete loss of private WSB/EiJ alleles in the central region flanked by selection markers, there was a concomitant loss of WSB/EiJ in the nearby flanking regions of the genome. The other seven founder haplotypes, although retained in the region targeted by the purge, no longer occur in the expected ratios due to either drift or inadvertent selection. The most substantial distortion across this region appears to be a reduced frequency of private NOD/ShiLtJ alleles. Earlier detection and reversal of segregation distortion in the DO population might have reduced this impact.

Deviations from idealized scenarios in MPPs are not insurmountable. For example, variation attributable to private WSB/EiJ variants on chromosome 2 is still amenable to study in the context of the CC and DO resources. The availability of a second population of outbred animals derived from the same founder strains, the HS-CC, provides an opportunity to query the impact of WSB/EiJ alleles in the *R2d2* region. It is interesting to note that the selective sweep in the HS-CC population was not complete by G25. This could reflect the impact of differences in the breeding scheme or reduction in frequency of genetic background effects that amplify drive at *R2d2*. WSB/EiJ alleles are also present in the CC inbred strains (Welsh *et al.* 2012). These resources will enable the identification of WSB/EiJ alleles with important phenotypic effects. Where questions of multi-locus interactions are concerned, strategies including CC × DO crosses, WSB/EiJ × DO crosses, and genome editing to reconstitute lost WSB/EiJ alleles in the DO population could be considered.

We suspected that changes in the WSB/EiJ allele frequency at the *R2d2* locus could affect litter size or sex ratio. We observed a decrease in litter size among *R2d2* heterozygous dams and an increase in nonproductive matings during the purge. Given the breeding design of the DO, the haplogroup frequencies for the mitochondria and Y chromosome should not change due to drift. However, selection in favor of particular breeders during the purge as well as against unproductive breeding pairs could have an effect. For the Y chromosome, we observe little effect of the *R2d2* purge. In contrast, there is a substantial effect on the mitochondria. We speculate that the strong reduction of the ABCD haplogroup frequency, in particular between G13 and G14, was caused by the overrepresentation of this haplotype in females that were either homozygous for the WSB allele at *R2d2* (and thus excluded from the matings) or had the high TRD phenotypes (and thus contributed fewer breeders to the next generation). We believe that this association was due to chance, as we do not find any evidence for an association between mitochondria haplotype and presence or level of TRD in the DO (data not shown).

We investigated other genomic features that could have been impacted by the purge, with particular focus on the sex chromosomes. It has been previously reported that the boundary of the pseudoautosomal region (PAR) of the sex chromosomes is located 430 kb proximal in the CAST/EiJ strain compared to other laboratory strains (White *et al.* 2012). In other words, this 430 kb interval is now present in both the X and Y chromosomes in CAST/EiJ, so the presence of heterozygosity in that region should be diagnostic for the presence of the CAST/EiJ Y chromosome. The X chromosome duplications in males described here extend further into the X chromosome beyond this “extended PAR” and are not exclusively associated with any specific Y chromosome haplogroup. Whether the duplications are X-linked, Y-linked, or pseudoautosomal, and whether they are associated with XO aneuploidy, is unknown. While the increased frequency of the duplication may be explained in part by the improved sensitivity to detect this type of abnormality with GigaMUGA (G21) as compared to MegaMUGA (G11), it is possible that biology is driving the accumulation of these duplications. The difference in the sizes of the duplicated segments and their prevalence among distributed DO mice ten generations apart suggest that these duplications are not rare in the DO population.

Multi-parent crosses have become increasingly popular for QTL studies (Gnan *et al.* 2014; Huang *et al.* 2014; Tsaih *et al.* 2014; Dell’Acqua *et al.* 2015) due to the idiosyncratic histories of existing reference populations and the desire to meet ideal properties of uniform allele frequencies, randomized assortment, and recombination. Here, we demonstrate the need for proactive monitoring and potential corrective measures to maintain the utility of populations like the DO. Although we tried to eliminate biases that are typically introduced in conventional breeding programs, this was not entirely possible and an unexpected, naturally occurring selection drove the population structure away from these ideals. In a randomized breeding scheme where synchronization of matings is required, mild selection against late reproductive maturity will also occur. Biological factors including meiotic drive, recombination hot spots, and other phenomena will ultimately dictate the uniformity of allele frequencies and mapping precision of the population. Despite our best efforts to eliminate the most obvious sources of selection bias in breeding, such events are unavoidable.

Our experience has provided several lessons for future efforts to construct multi-parent reference populations. Population monitoring is a crucial aspect of the development of genetic populations. It is essential for designers of MPPs to clearly prioritize resource development relative to the interesting research made possible by the study of undisrupted breeding history. Prior definition of quality metrics by stakeholders, *e.g.*, acceptable range of segregation distortion, long range linkage disequilibrium, and attrition due to reproductive variability, can facilitate rapid remedial measures to preserve essential population characteristics. In the case of the DO, remediation of segregation distortion to restore the population to its utility as a resource required a deviation from the original breeding plan and the noninterventionist ethos of its stakeholders. All multi-parent genetic reference populations deviate from the ideal in some sense. Selection and TRD are inevitable. However, with careful monitoring and selective interventions, the essential character of the population—balanced allele frequencies and low average kinship—can be retained. What is required for any population is a set of reliable lineage specific genotyping primers for each cross progenitor, routine cost-effective monitoring of cross-sectional genome-wide deviation from expected allele frequencies, and a sufficiently rapid genotyping and breeding decision process to enable marker-assisted interventions. It is possible that other distortions could emerge as DO production continues. We have instituted a routine check on allele frequencies across the population, and will be able to rapidly detect and evaluate the need for future interventions. By carefully monitoring allele frequencies we can detect and reverse unexpected changes and preserve the value of the DO as a premier platform for systems genetics in mammals, and recommend proactive monitoring and intervention in the construction of multi-parent resources.

## Supplementary Material

Supplemental material is available online at www.g3journal.org/lookup/suppl/doi:10.1534/g3.116.035527/-/DC1.
