# Supplementary figures and images for: Diversity Outbred Mice at 21: Maintaining Allelic Variation in the Face of Selection
Source: G3 (Bethesda). 2016 Sep 29;6(12):3893–902. doi: 10.1534/g3.116.035527 (PMC5144960; doi:10.1534/g3.116.035527)

CHR 1

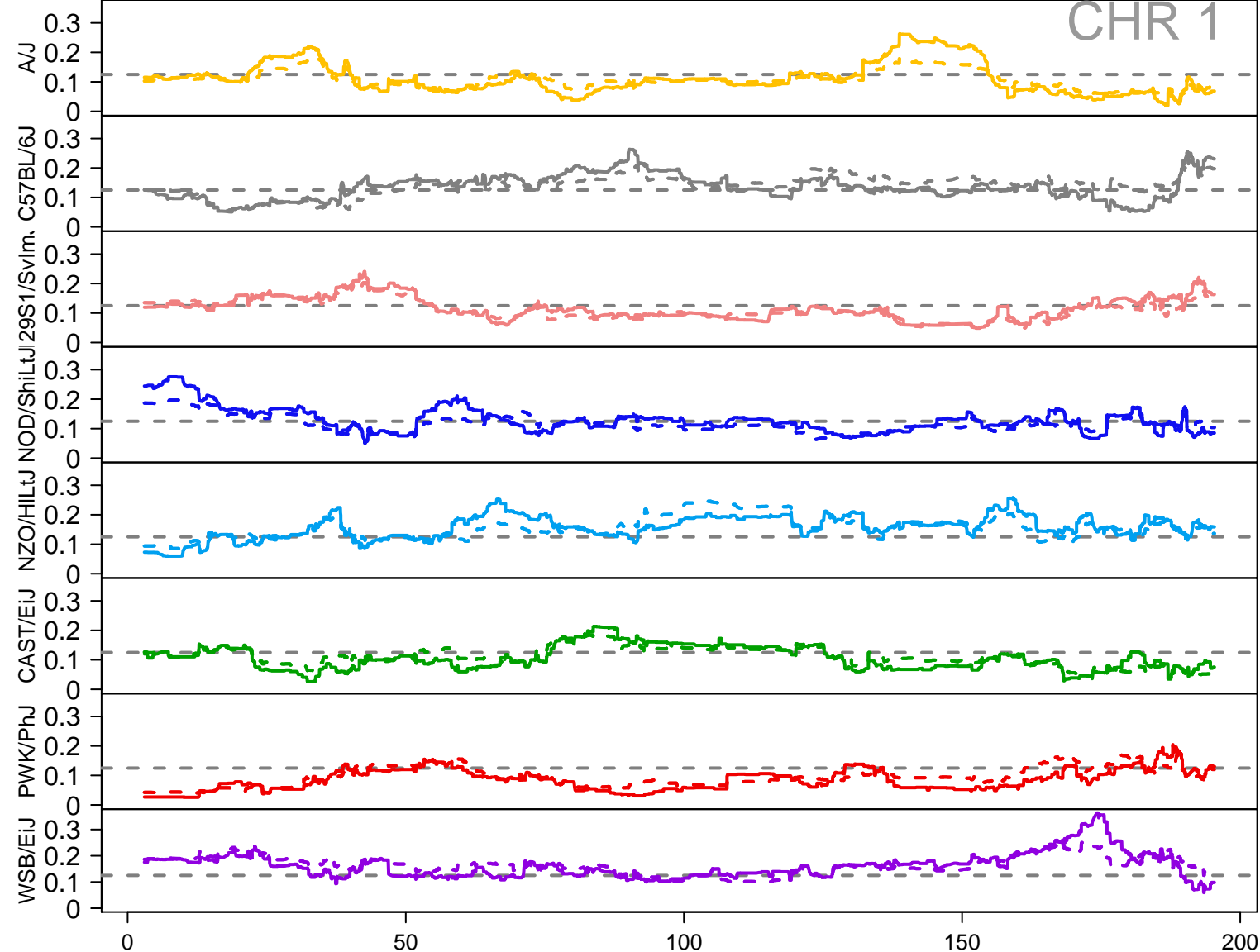

CHR 2

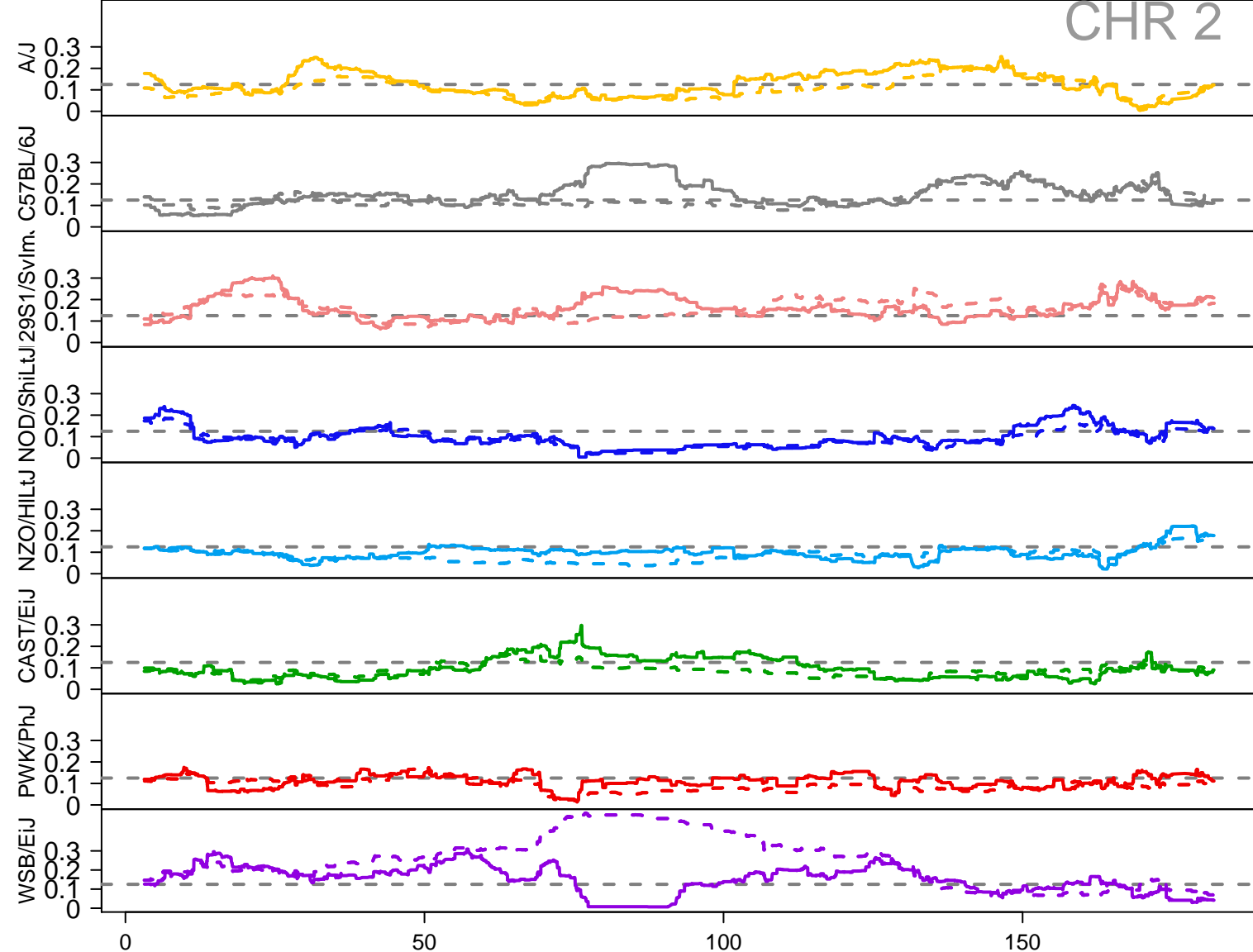

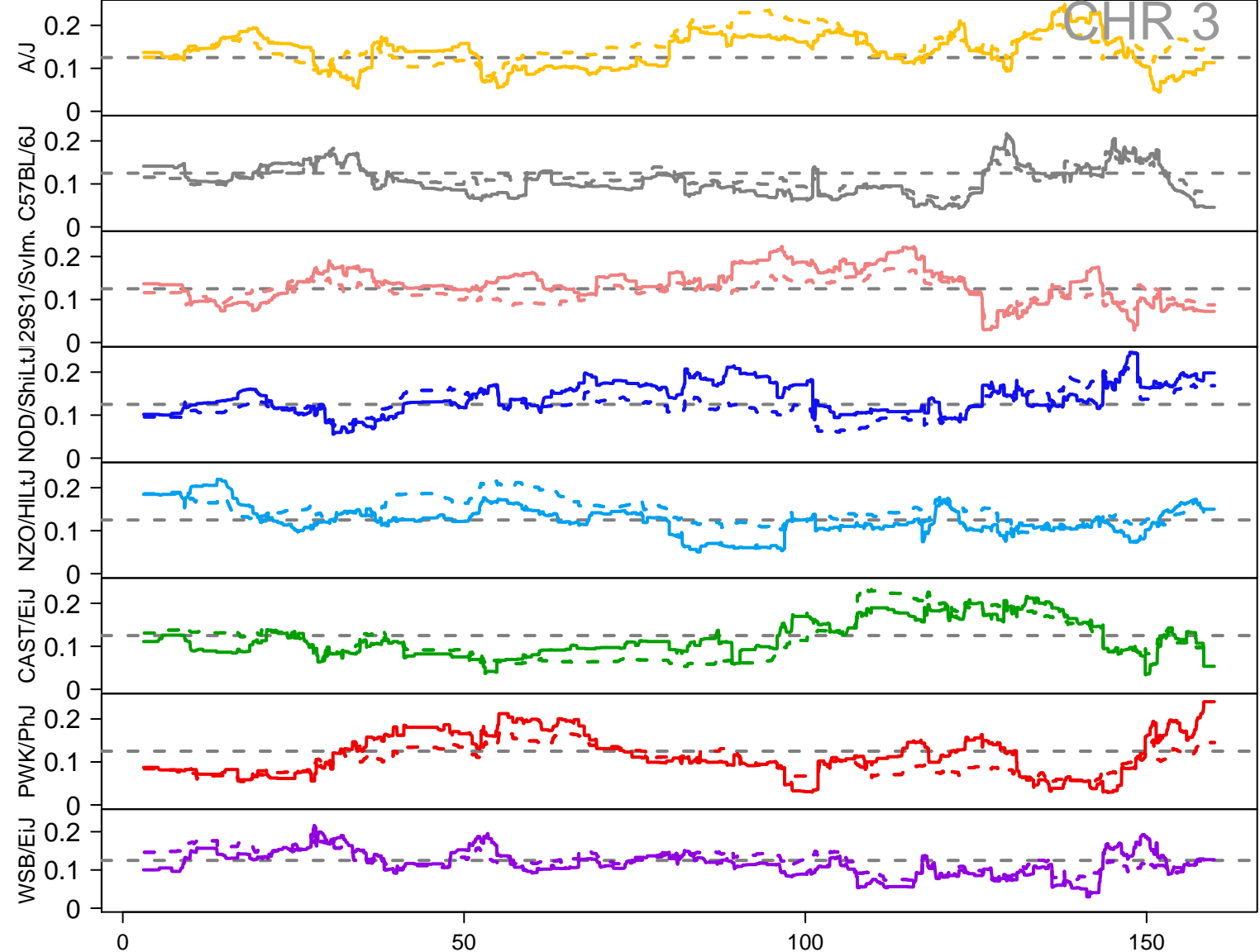

CHR 4

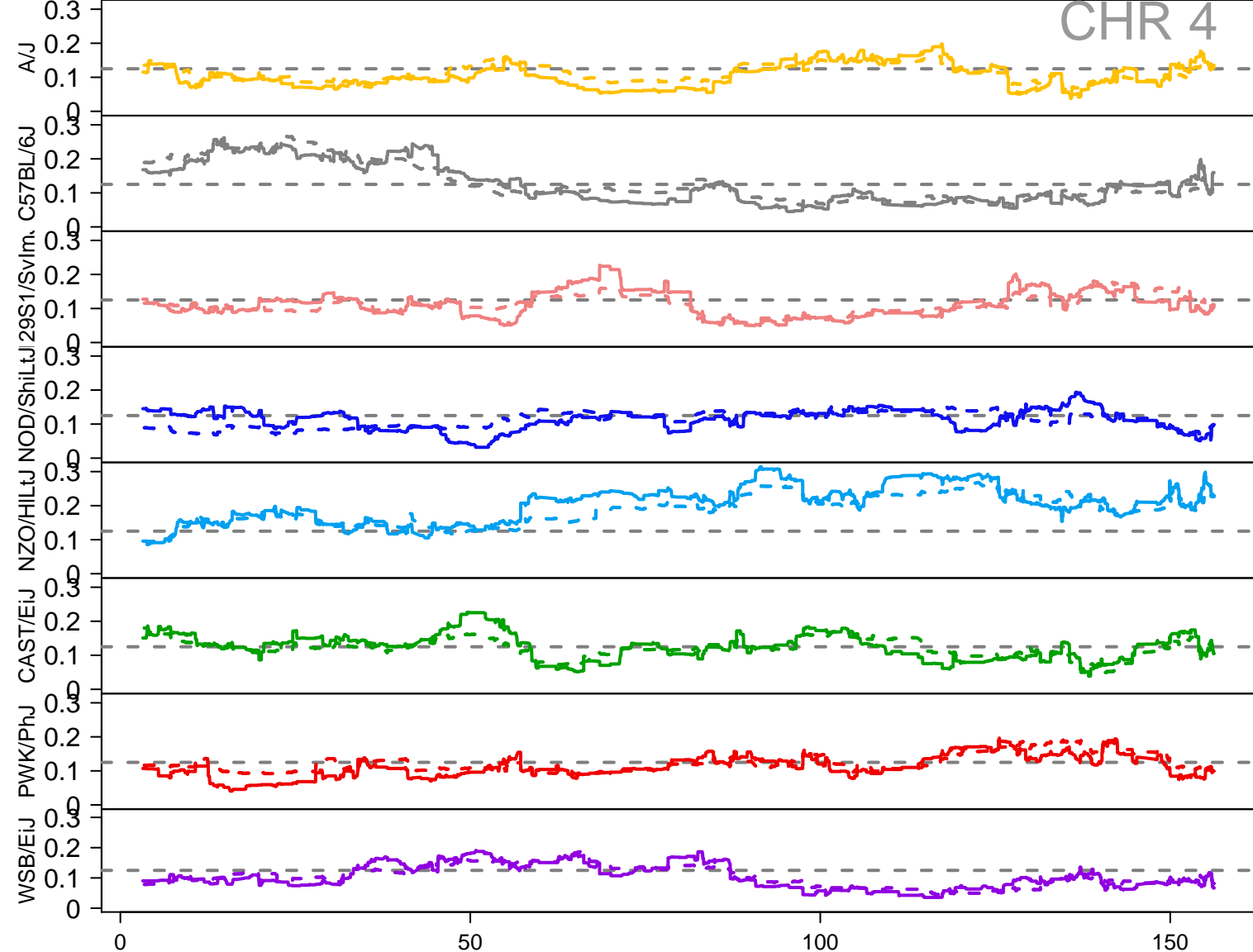

CHR 5

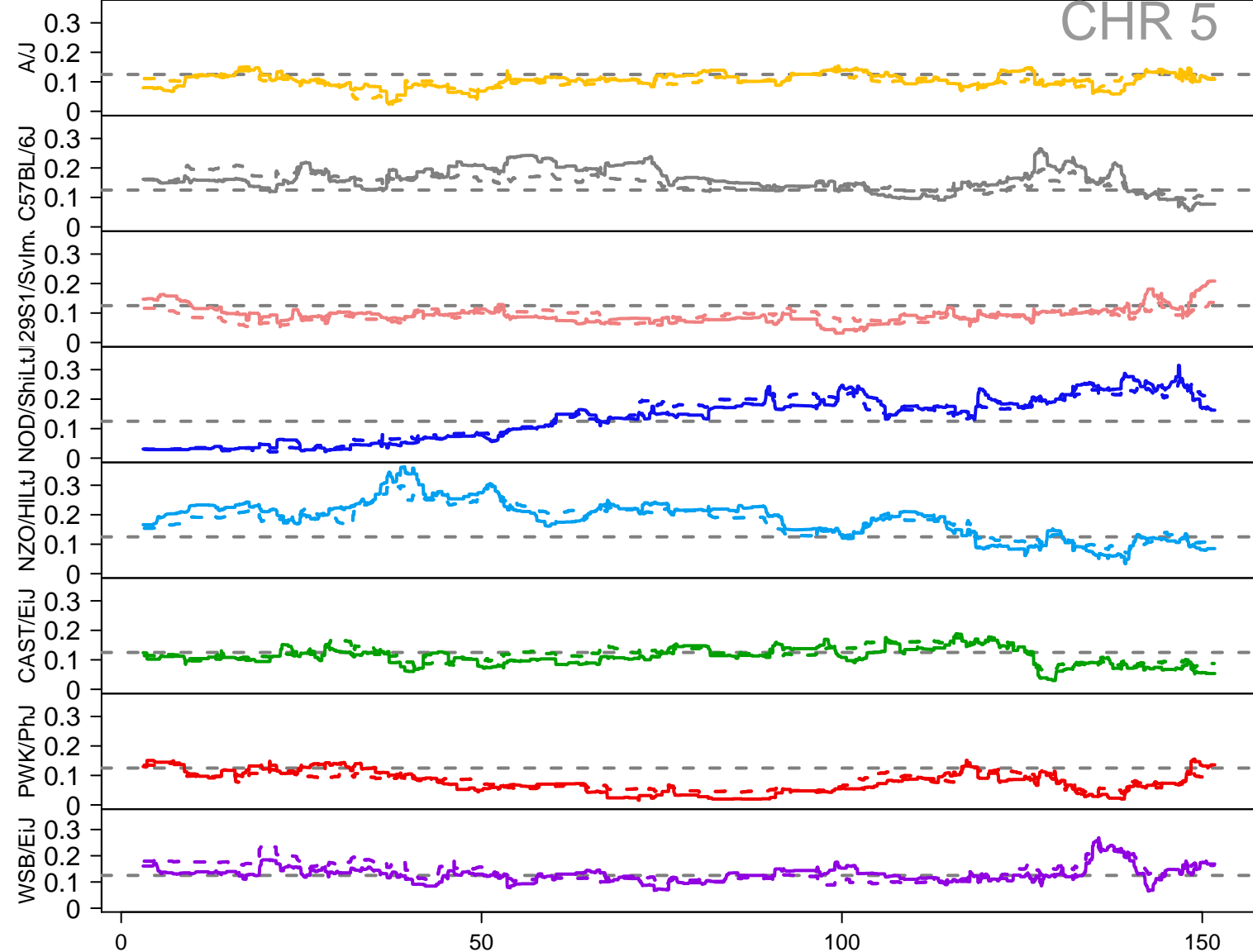

CHR 6

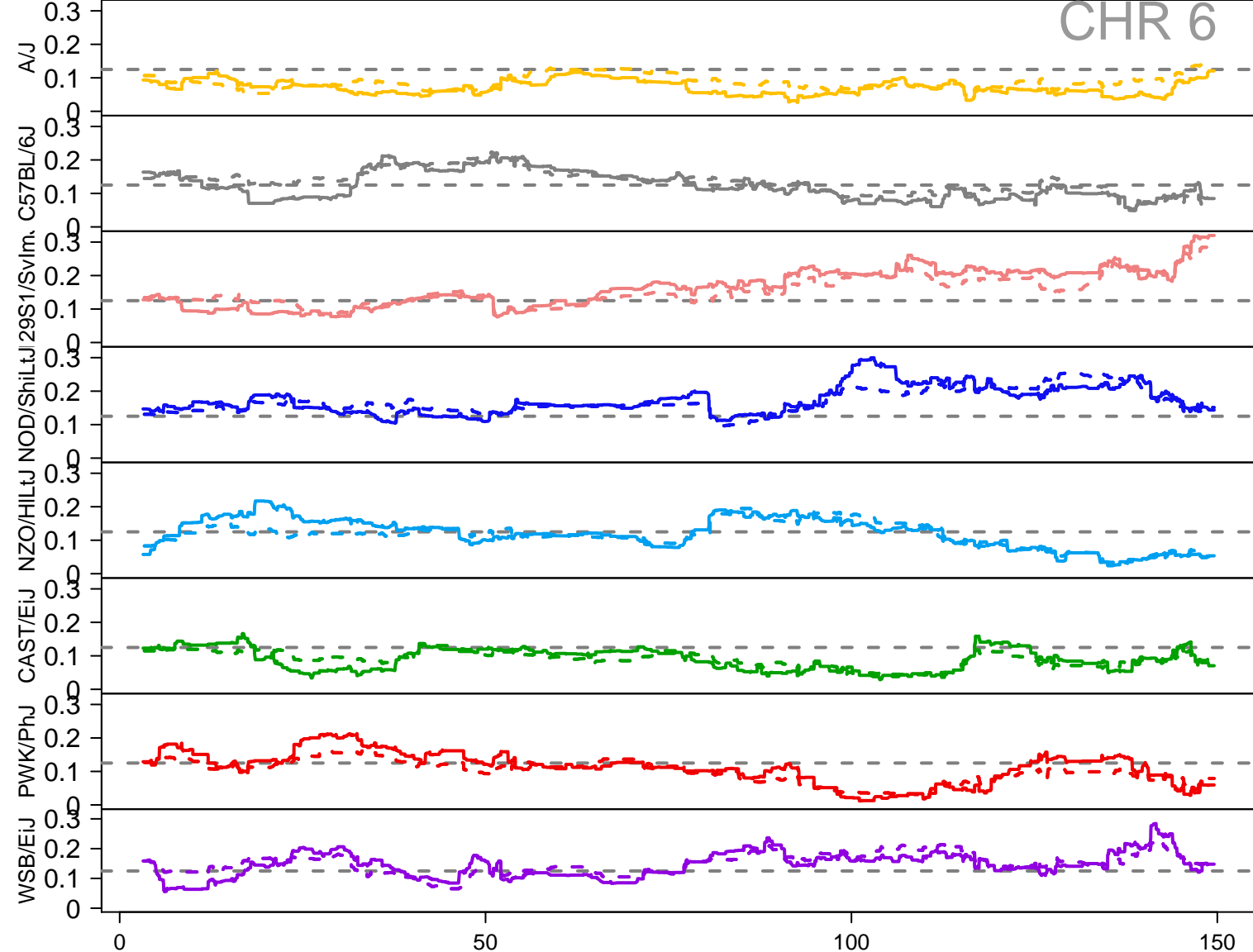

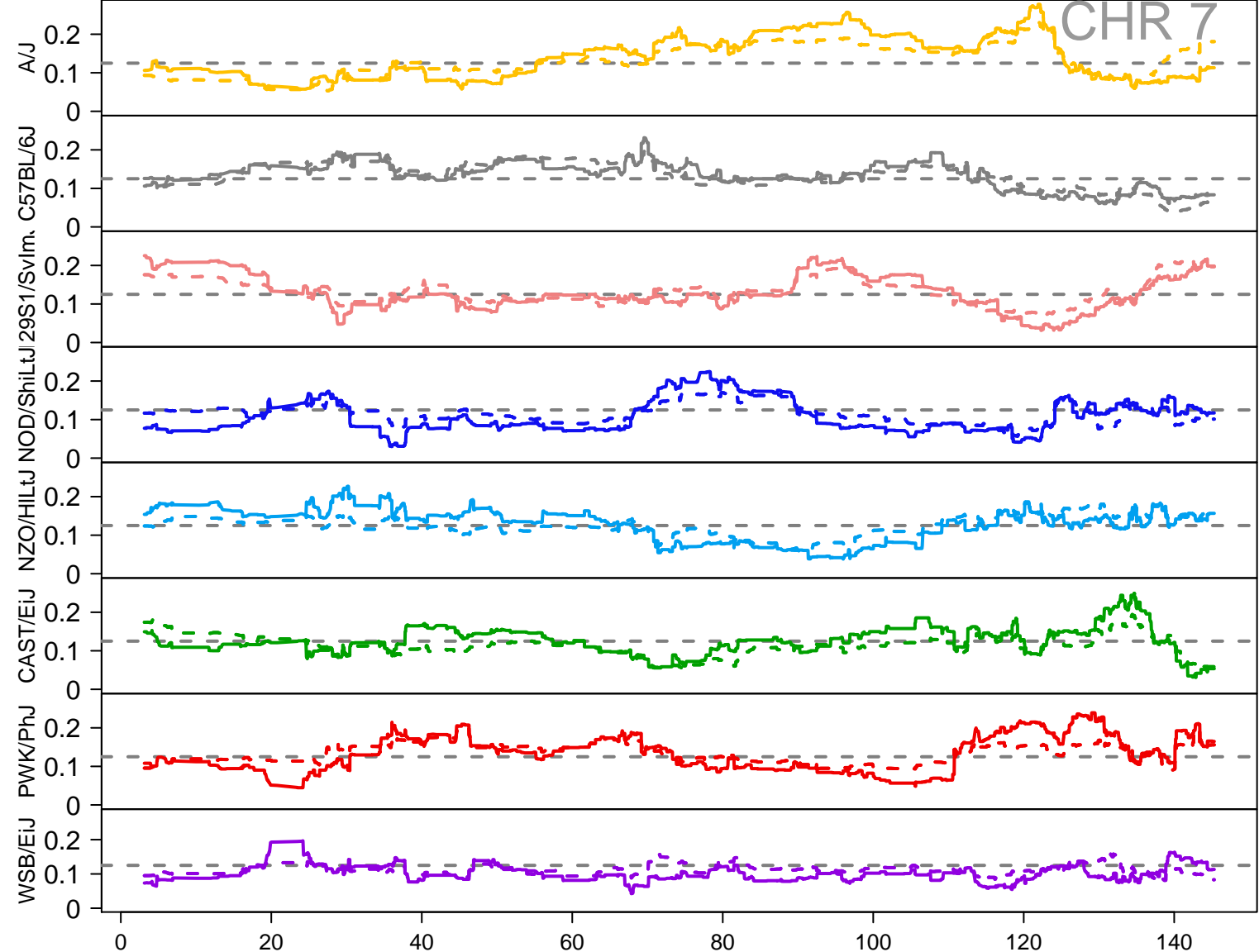

CHR 8

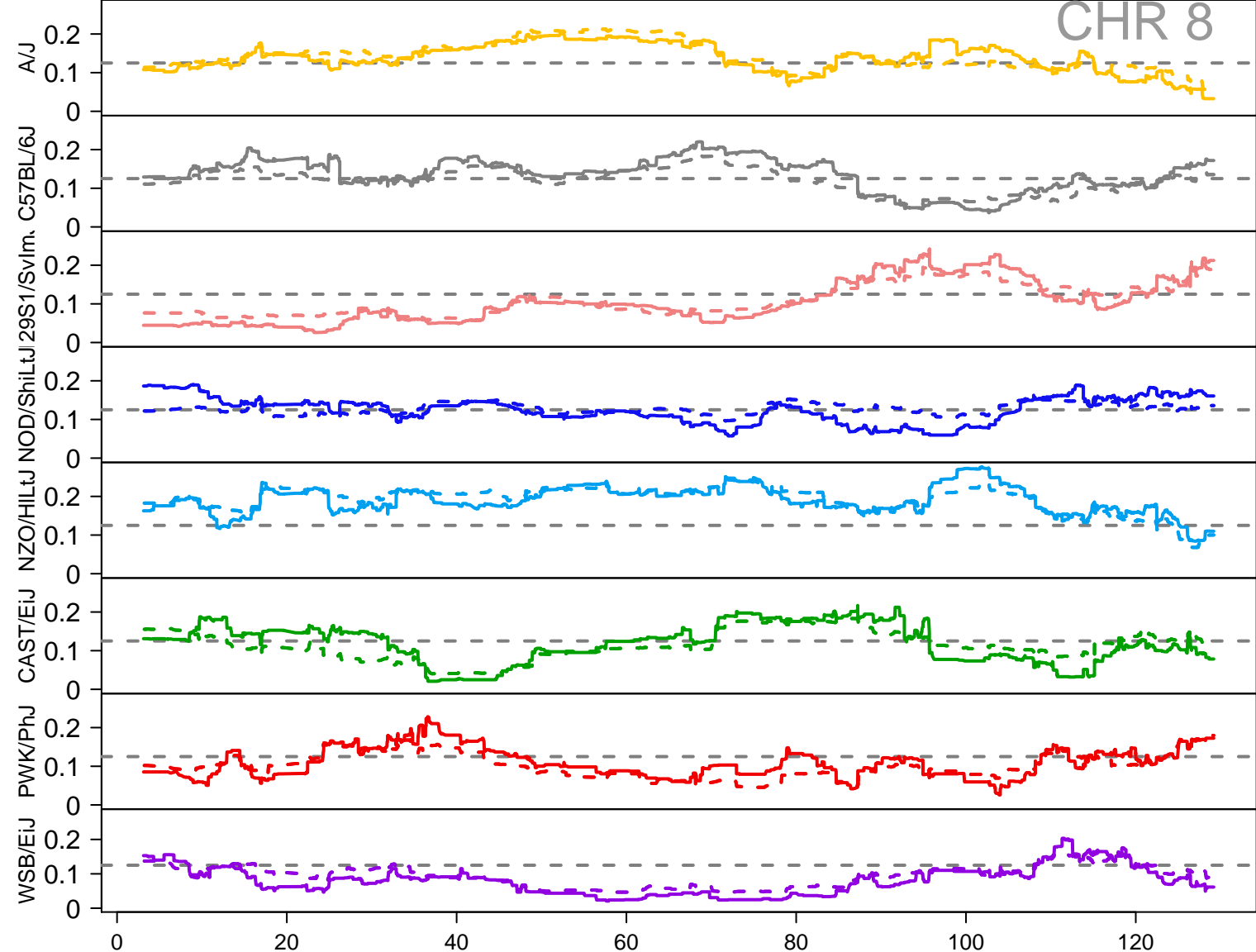

CHR 9

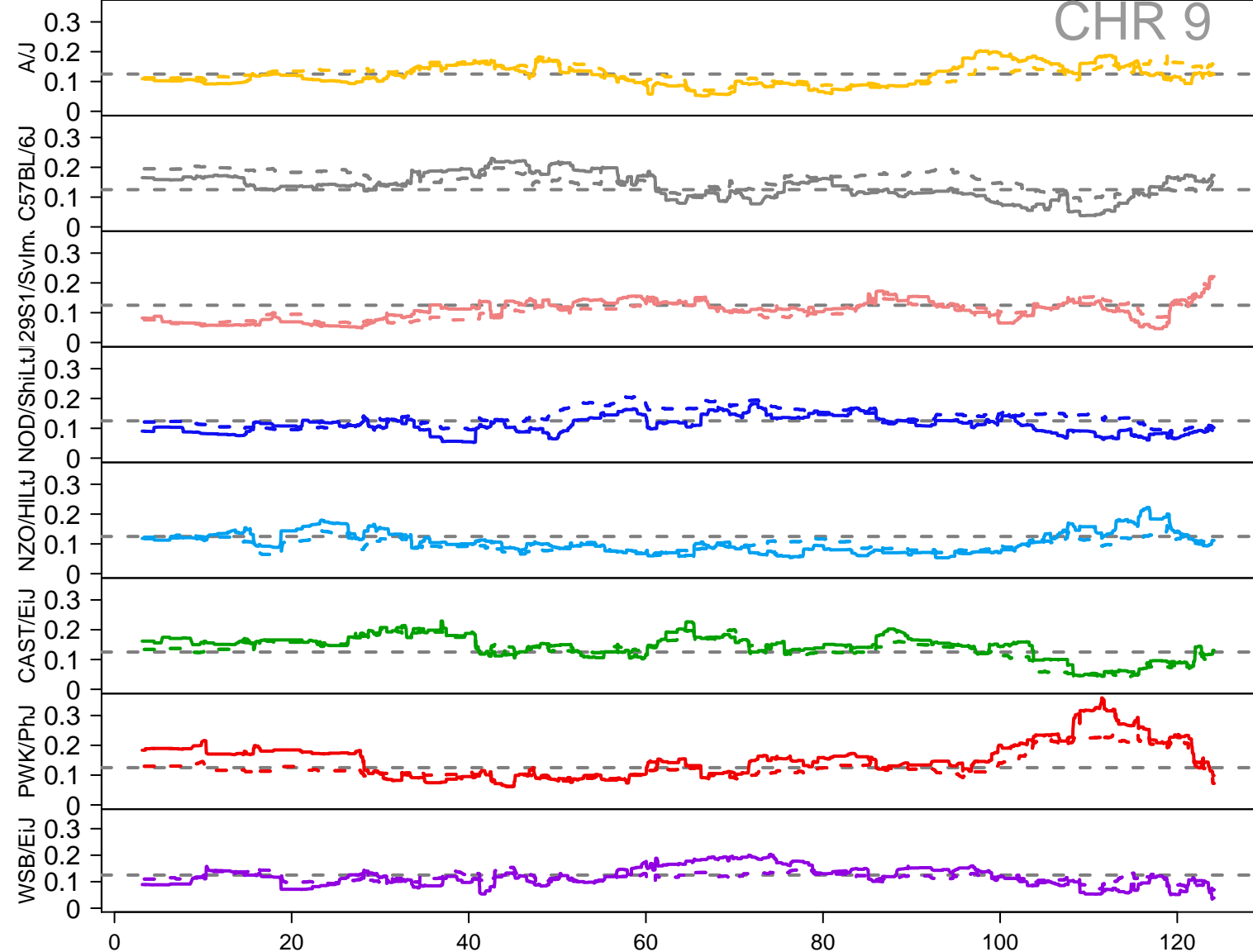

CHR 10

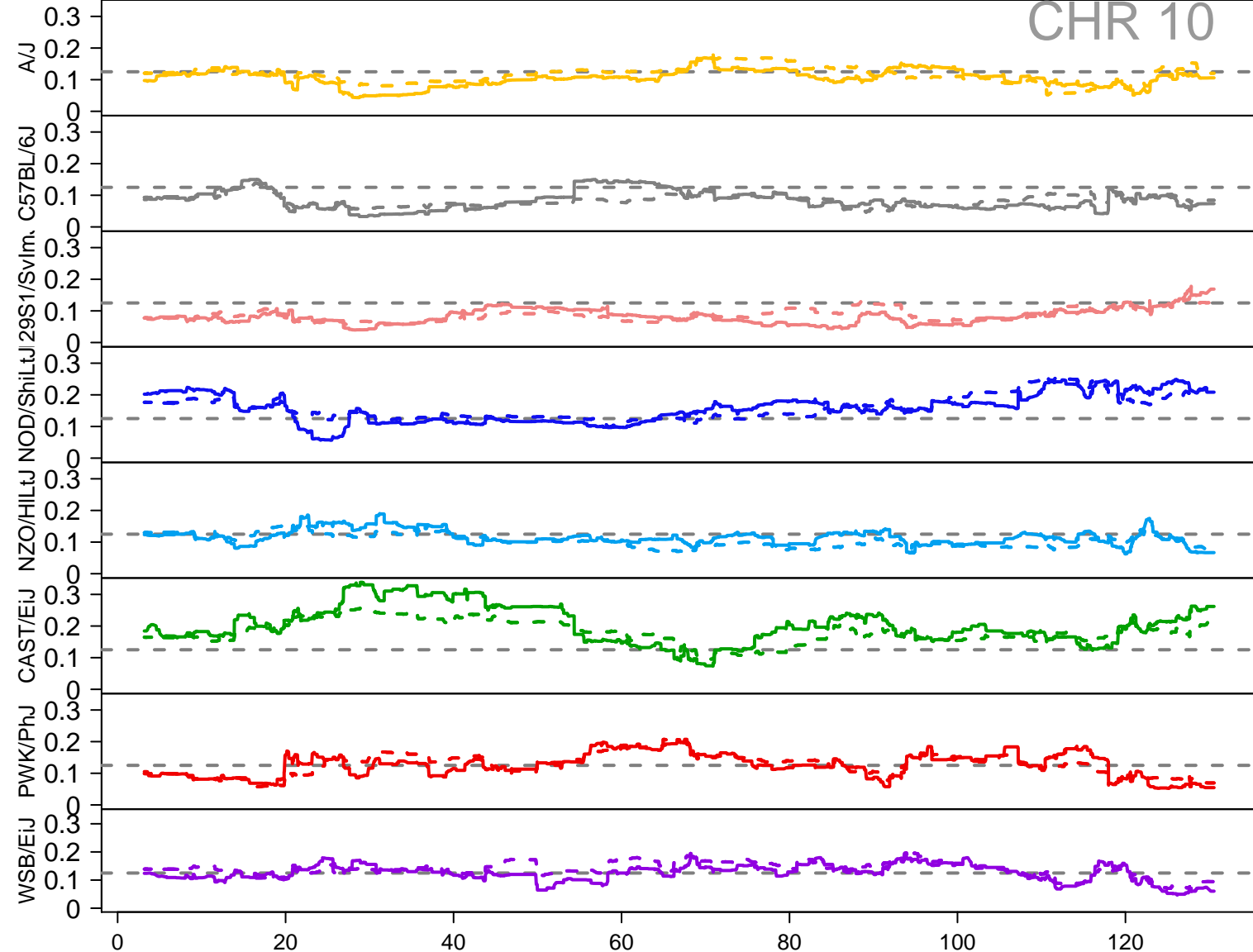

CHR 11

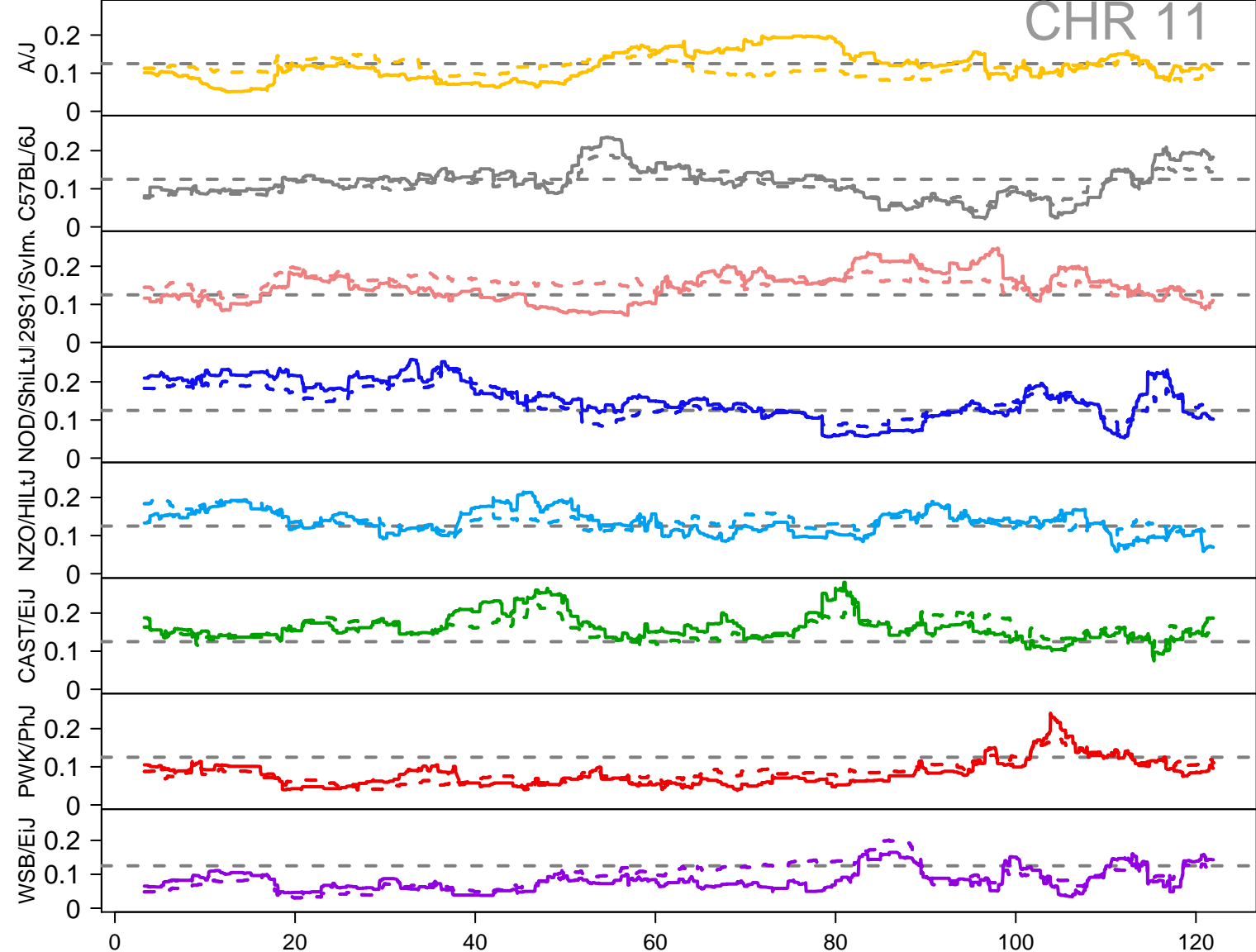

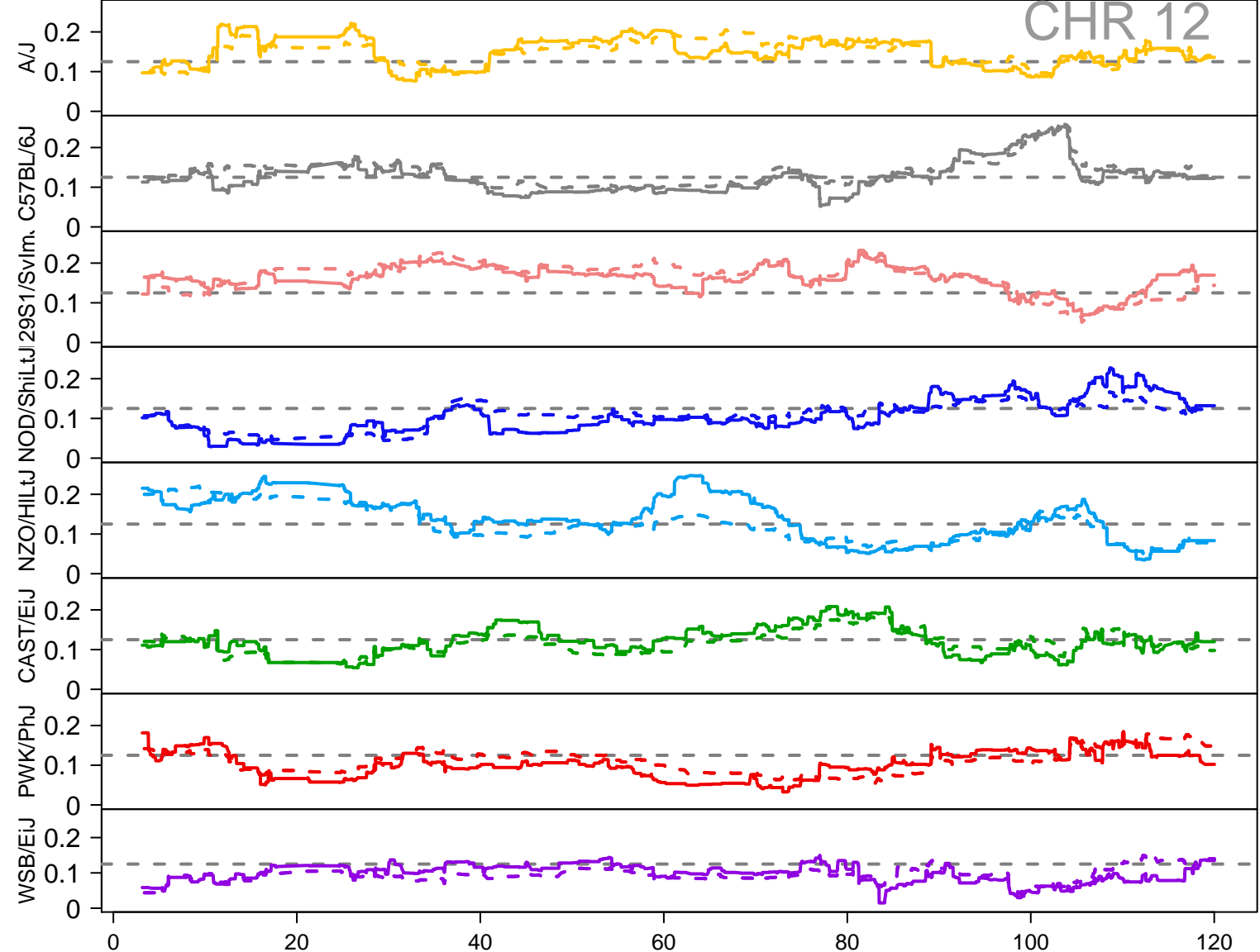

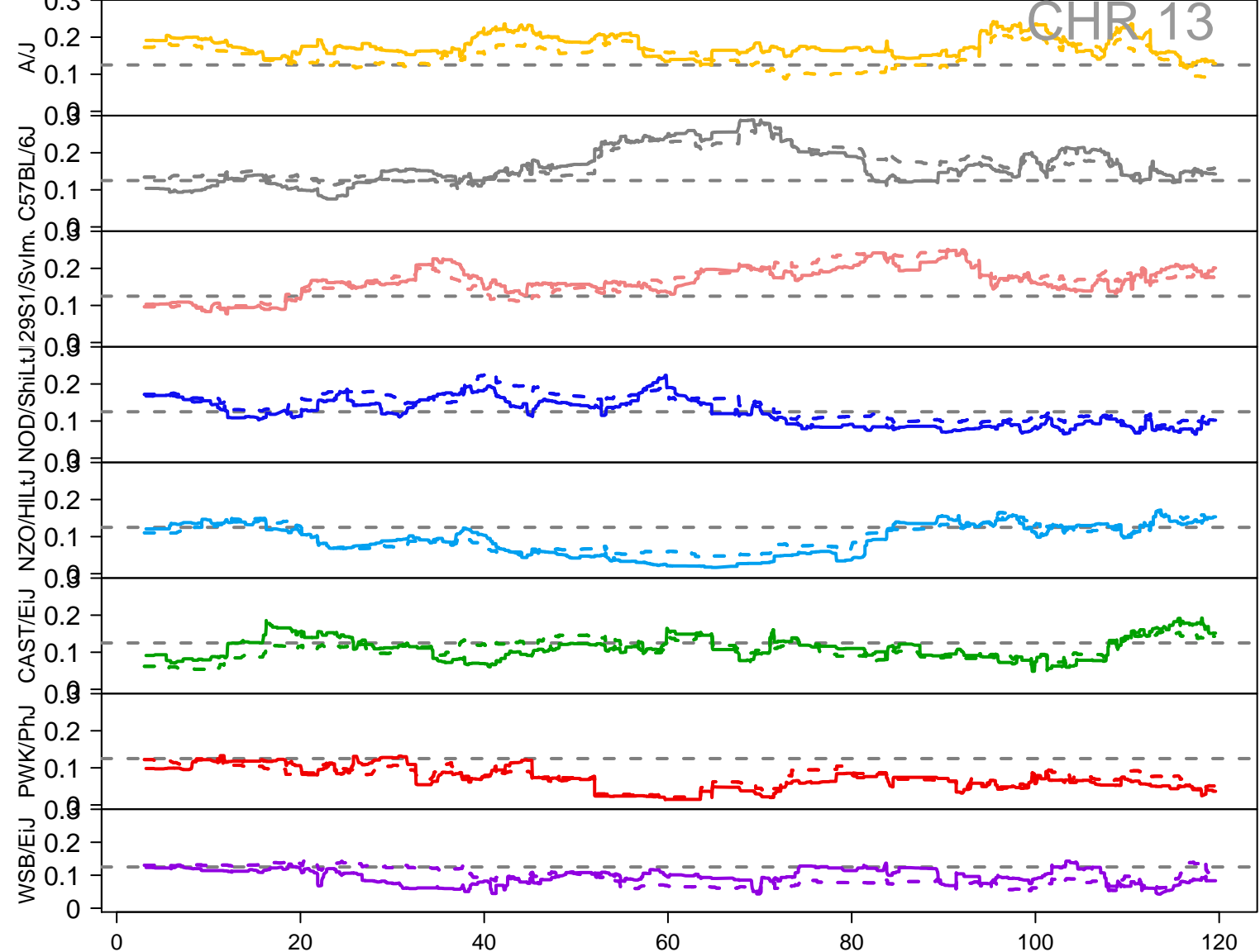

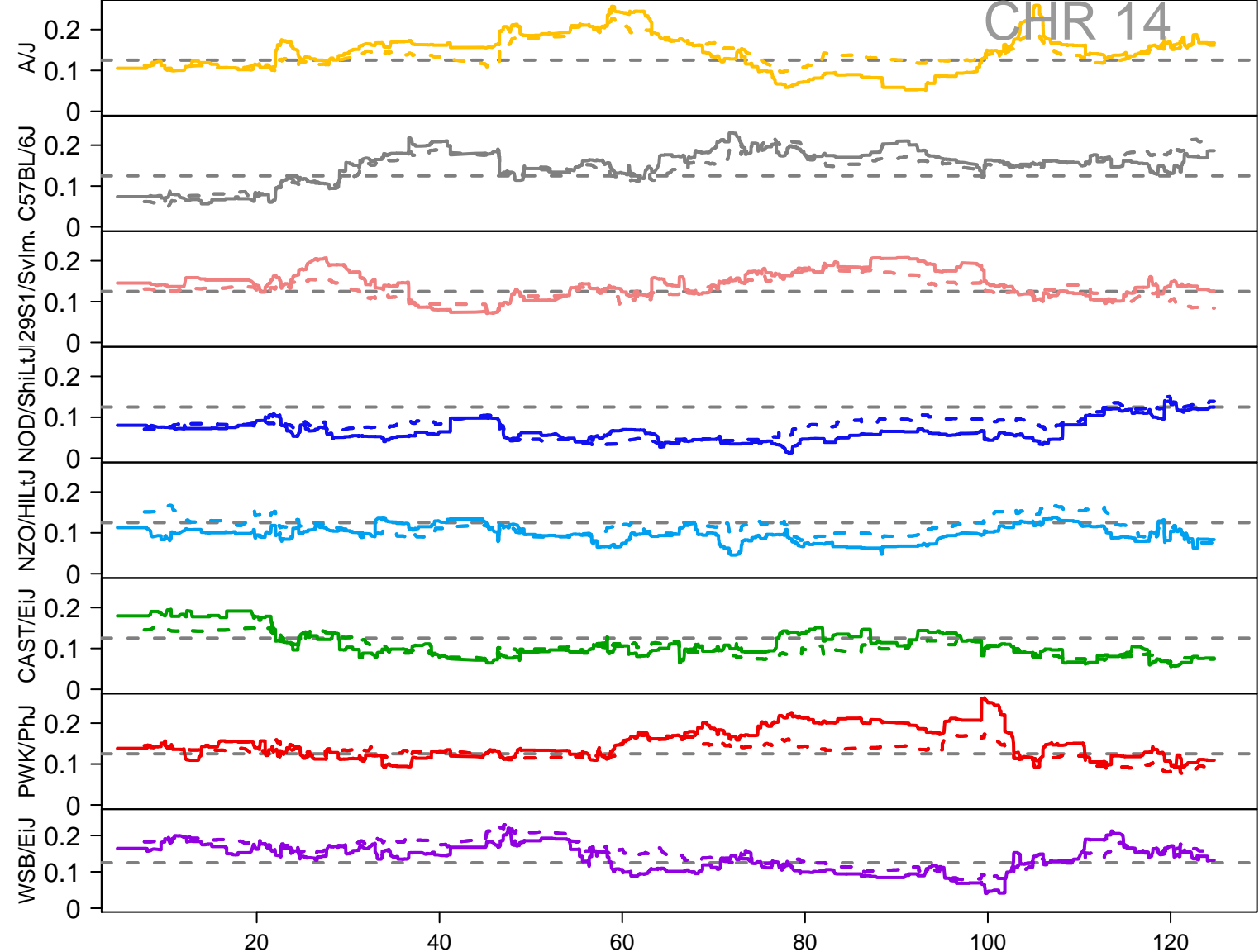

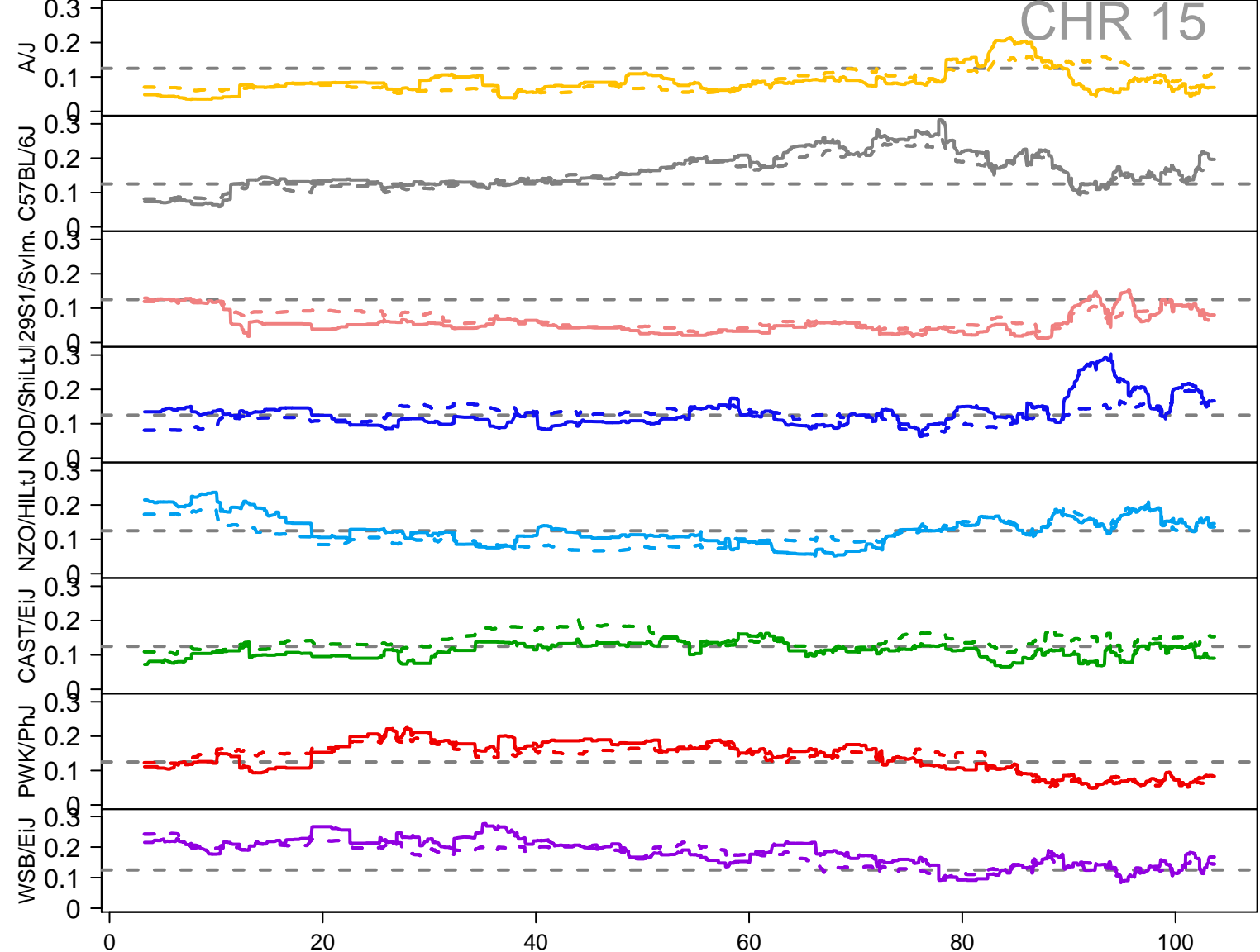

CHR 16

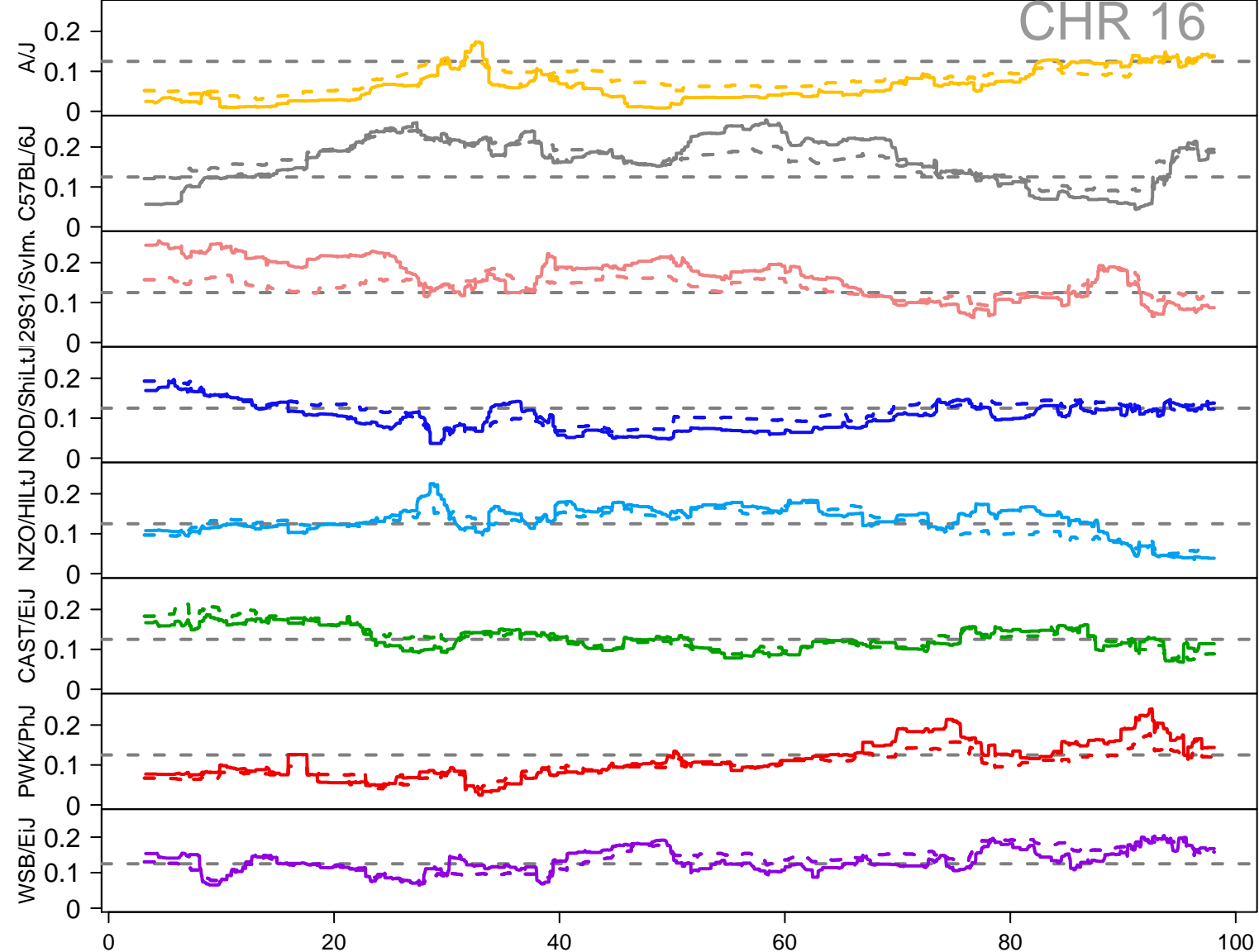

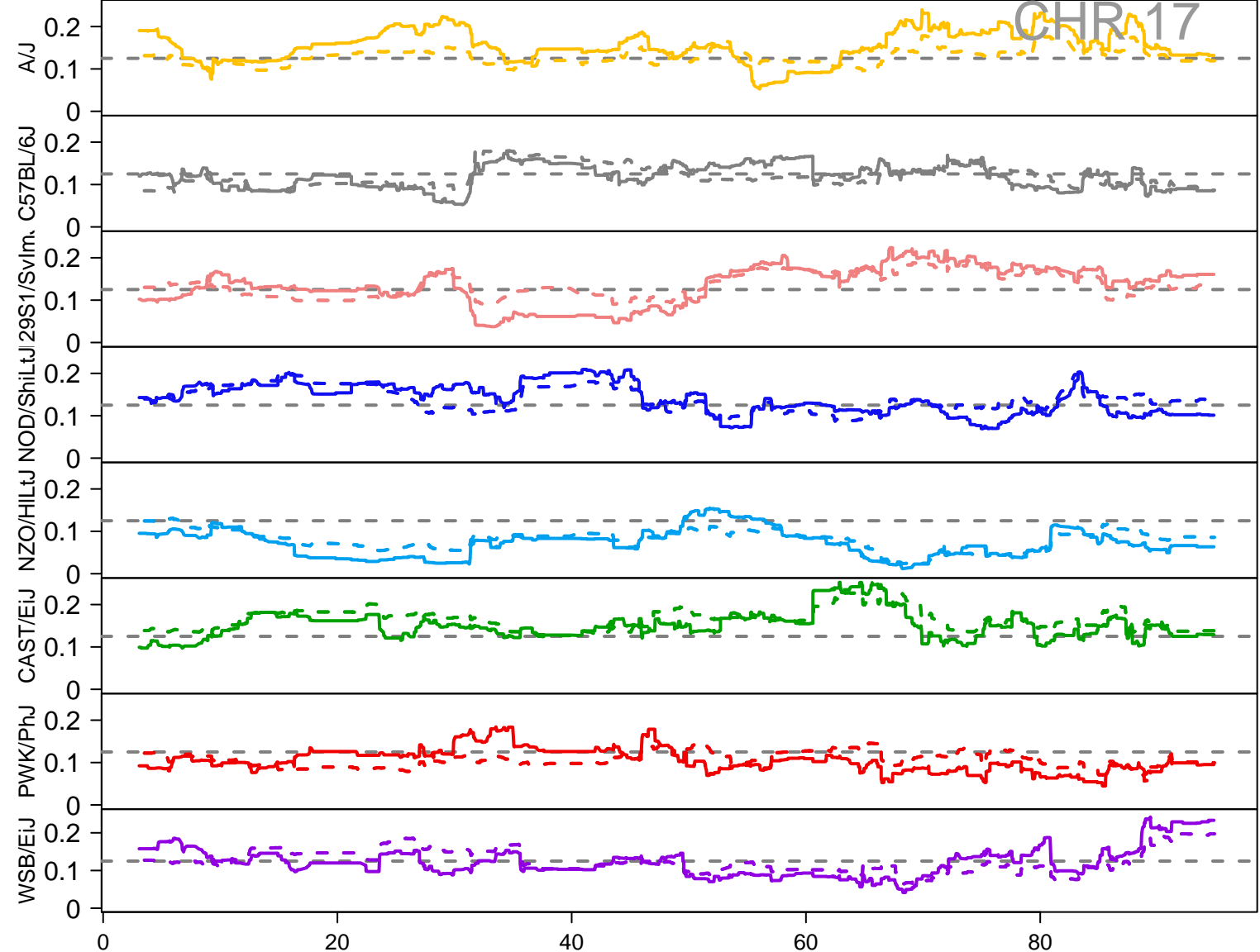

CHR 18

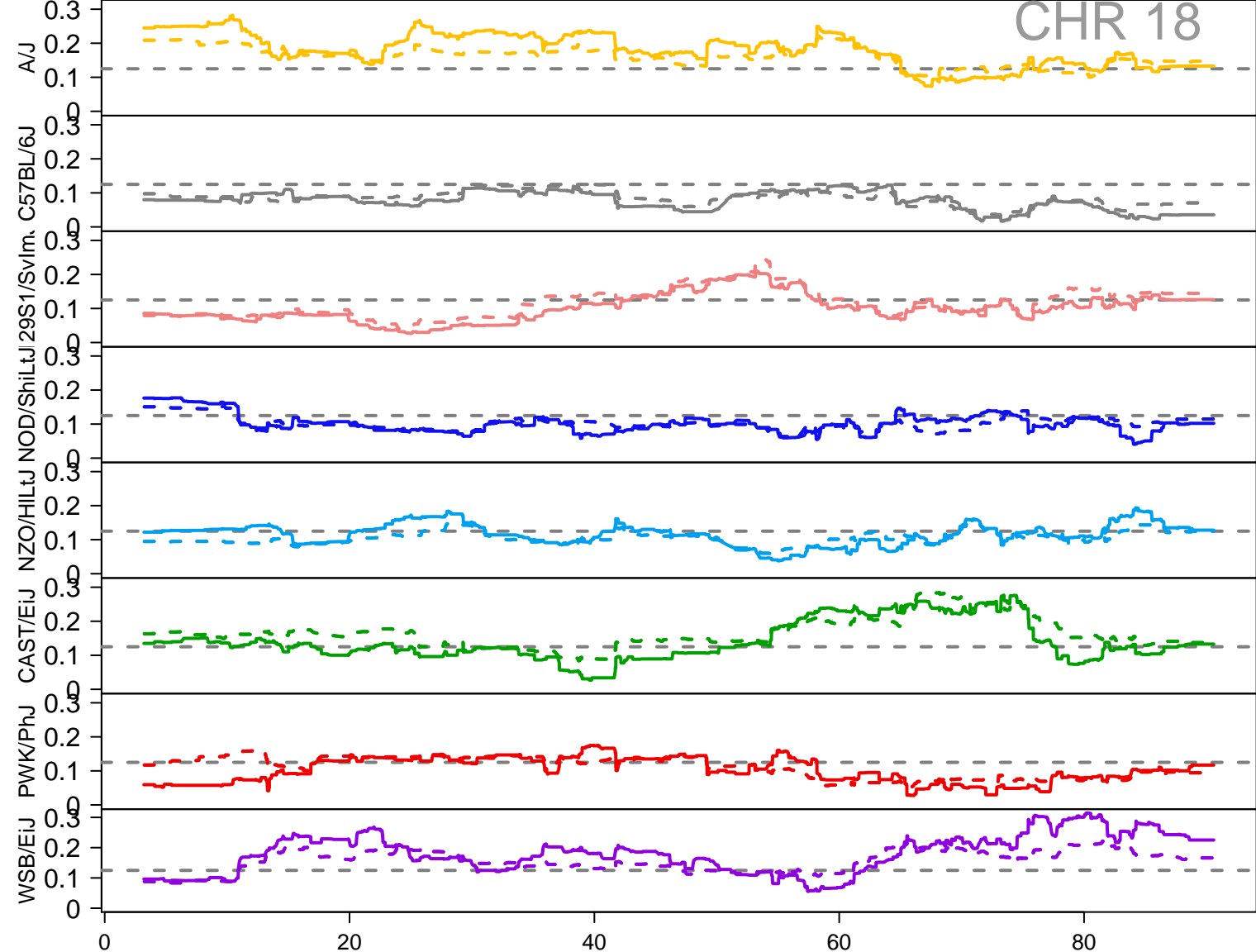

CHR 19

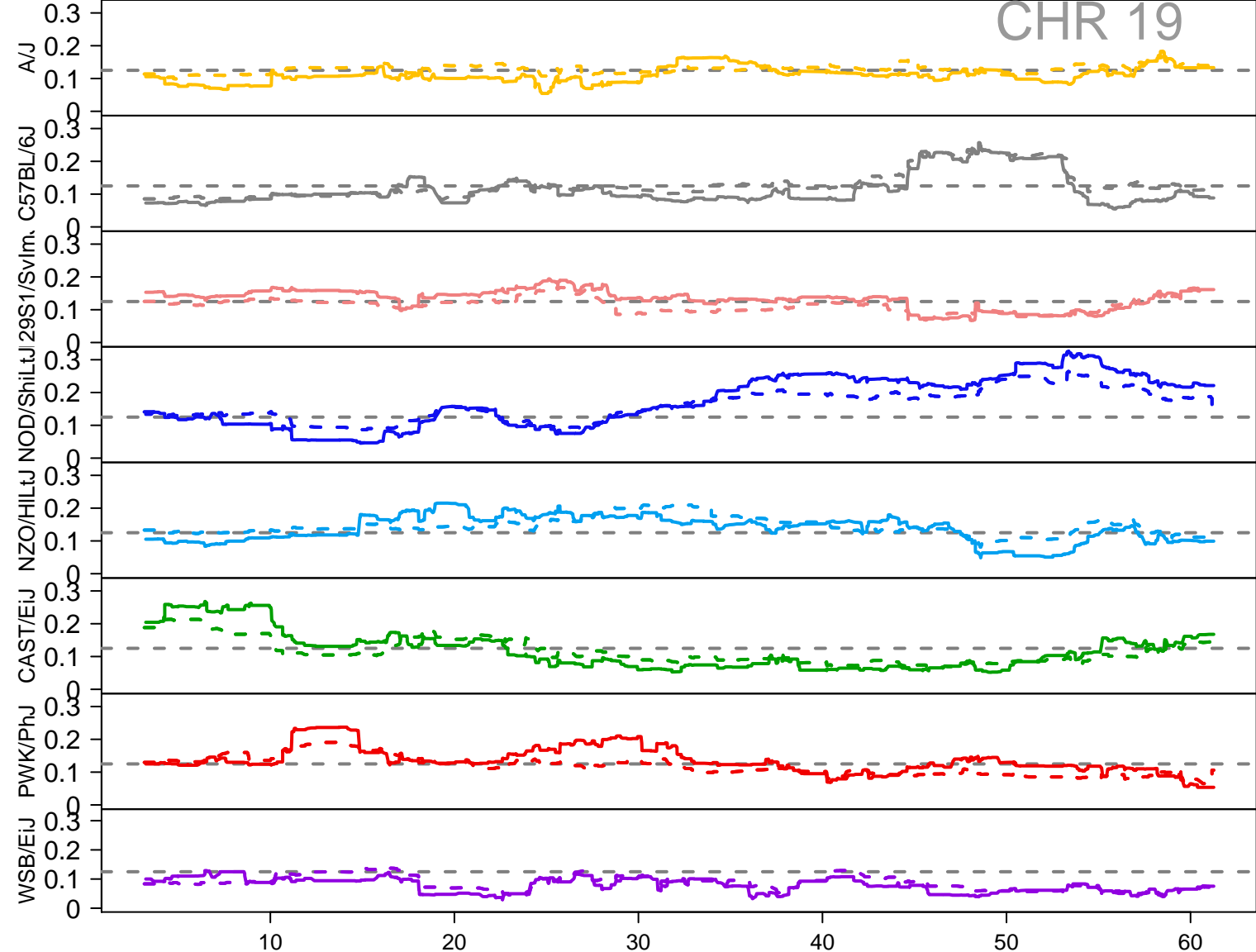

CHR X

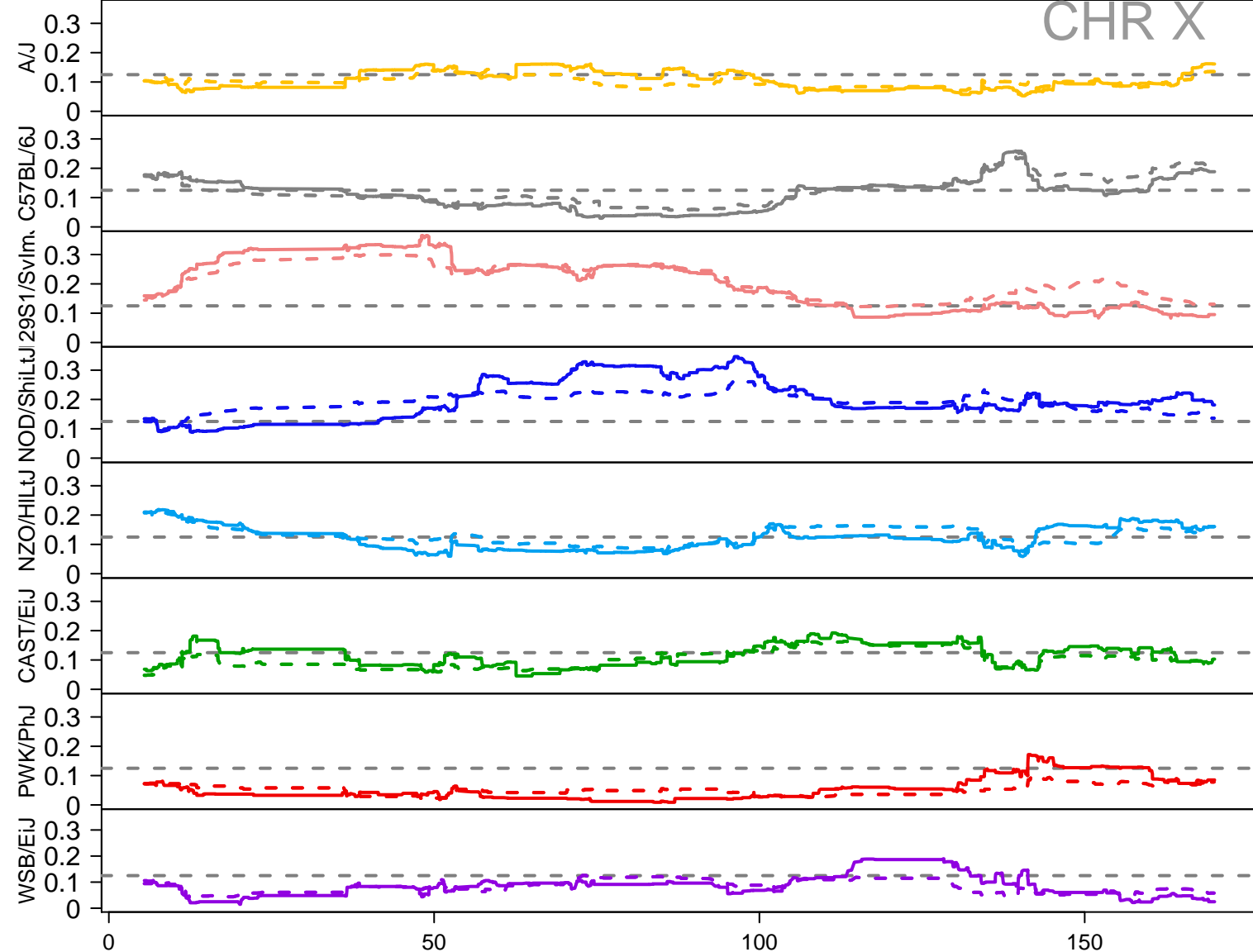

Supplement: Supplementary file 3 [file 3893FileS3.pdf]

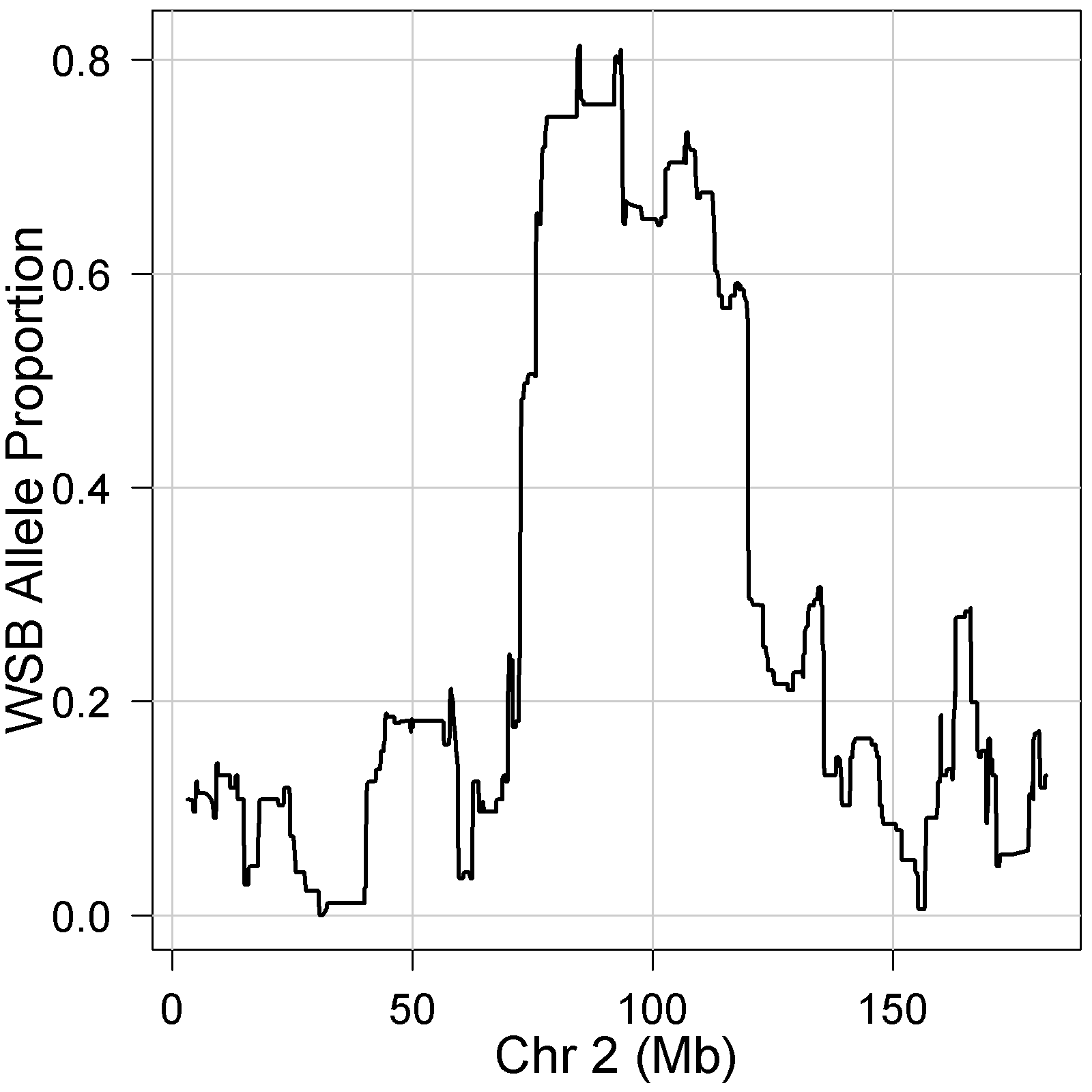

Supplement: Supplementary file 6 [file 3893FigureS1.png]
