# Supplementary material for: Diversity Outbred Mice at 21: Maintaining Allelic Variation in the Face of Selection
Source: G3 (Bethesda). 2016 Sep 29;6(12):3893–902. doi: 10.1534/g3.116.035527 (PMC5144960; doi:10.1534/g3.116.035527)
Supplement: Supplementary file 4 [file 3893FileS4.pdf]

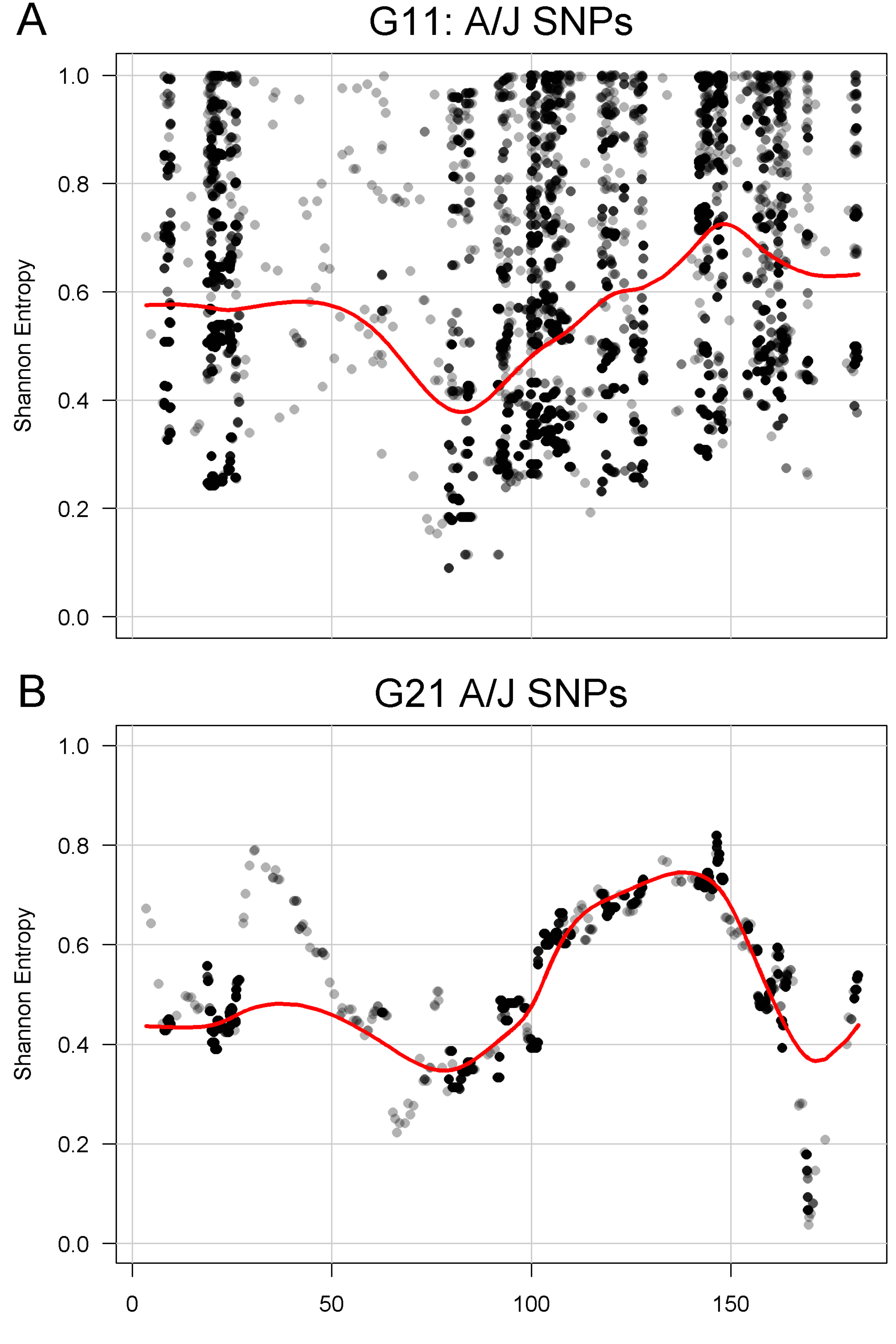
A
G11: A/J SNPs
Shannon Entropy
1.0
0.8
0.6
0.4
0.2
0.0
B
G21 A/J SNPs
Shannon Entropy
1.0
0.8
0.6
0.4
0.2
0.0
0
50
100
150

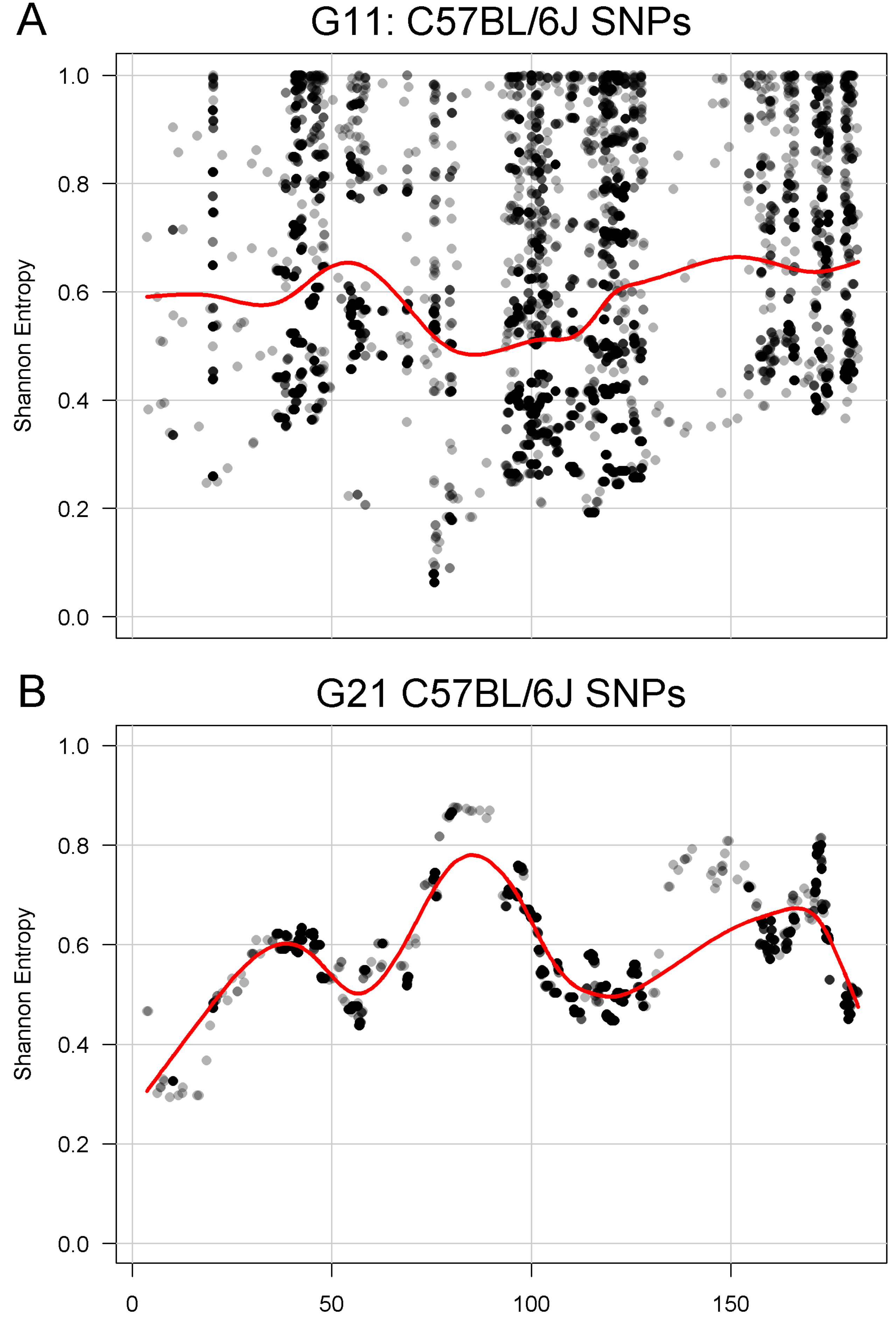
A
G11: C57BL/6J SNPs
Shannon Entropy
1.0
0.8
0.6
0.4
0.2
0.0
B
G21 C57BL/6J SNPs
Shannon Entropy
1.0
0.8
0.6
0.4
0.2
0.0
0
50
100
150

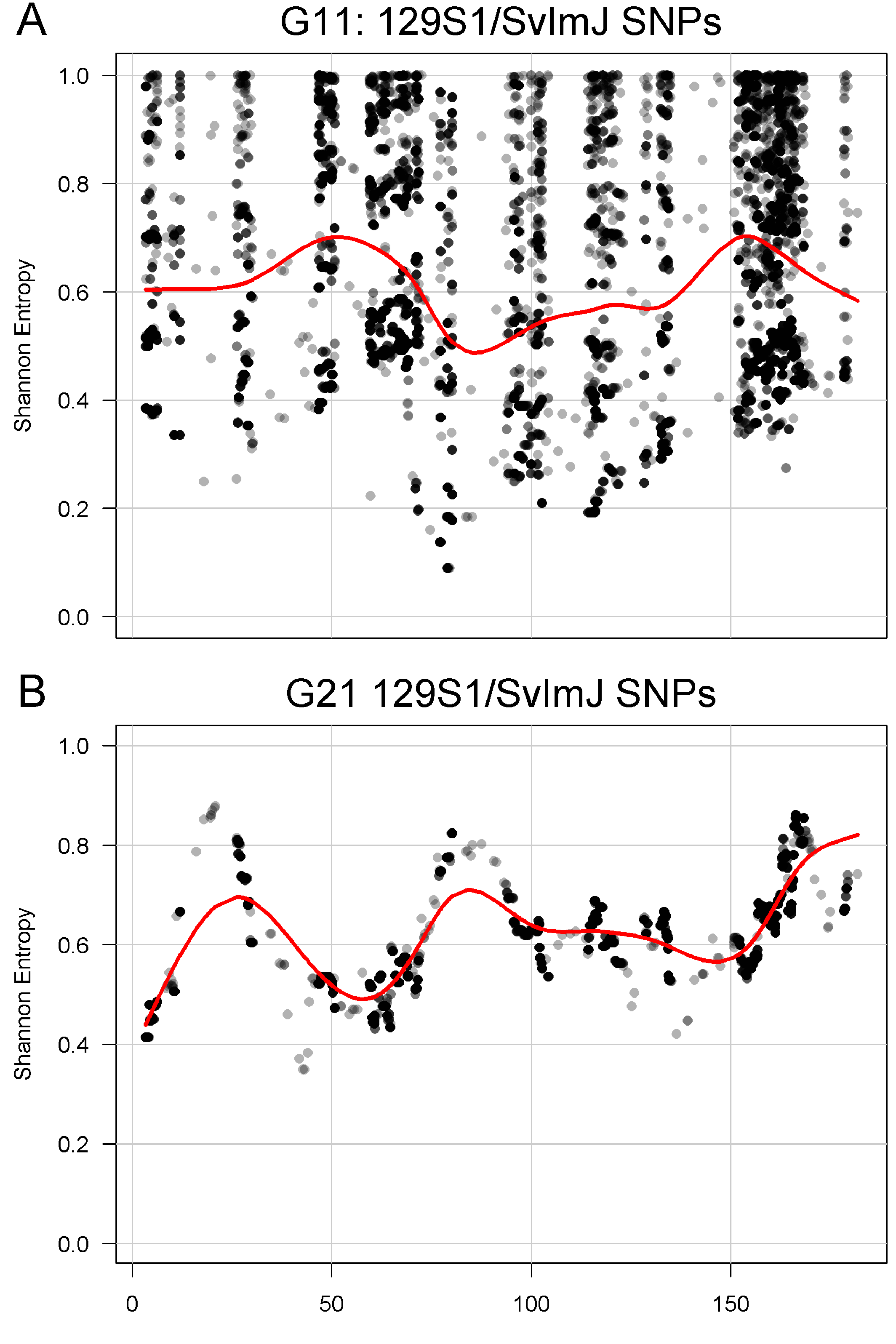

A
G11: 129S1/SvImJ SNPs
Shannon Entropy
0.0
0.2
0.4
0.6
0.8
1.0
B
G21 129S1/SvImJ SNPs
Shannon Entropy
0.0
0.2
0.4
0.6
0.8
1.0
0
50
100
150

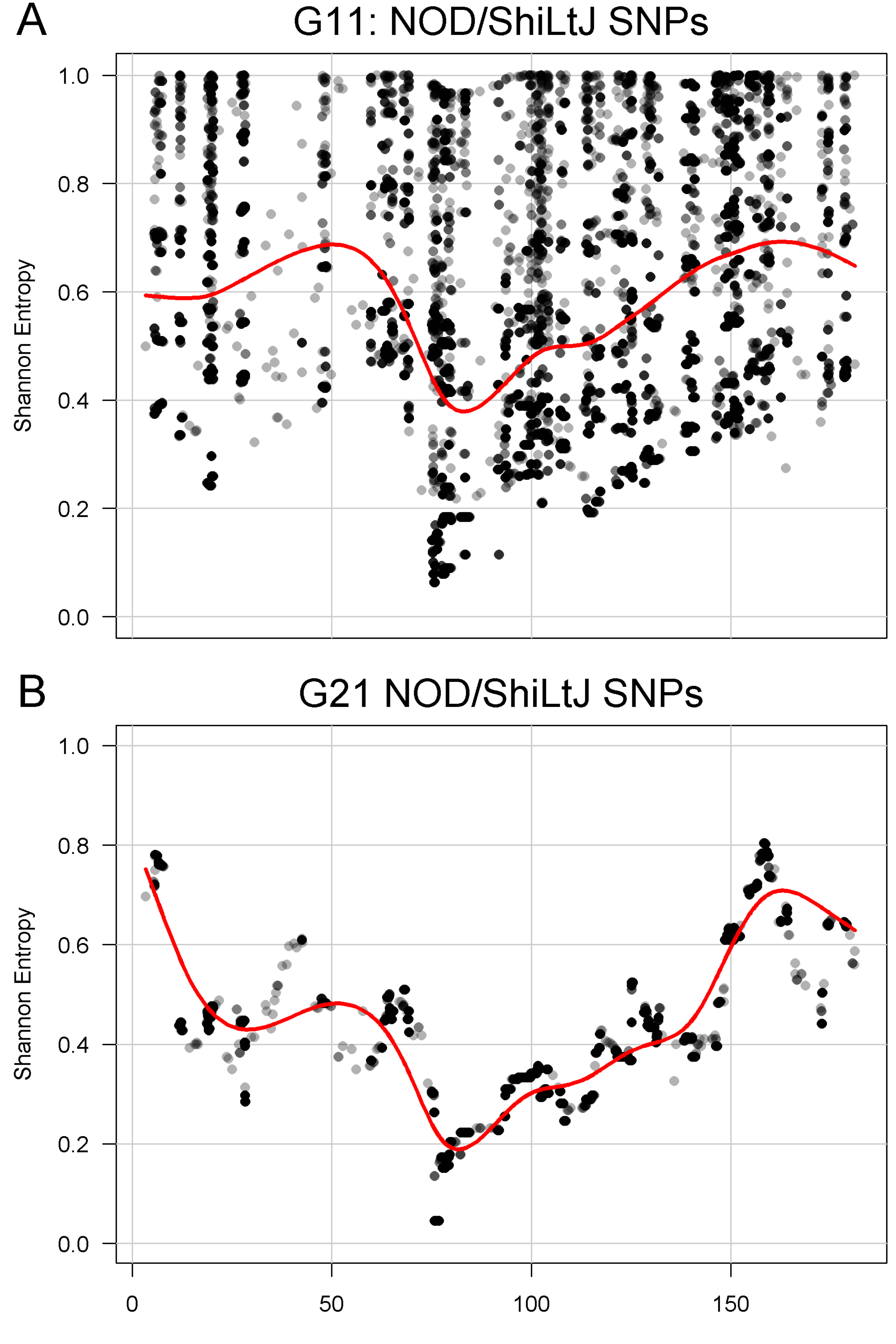
A
G11: NOD/ShiLtJ SNPs
Shannon Entropy
1.0
0.8
0.6
0.4
0.2
0.0
B
G21 NOD/ShiLtJ SNPs
Shannon Entropy
1.0
0.8
0.6
0.4
0.2
0.0
0
50
100
150

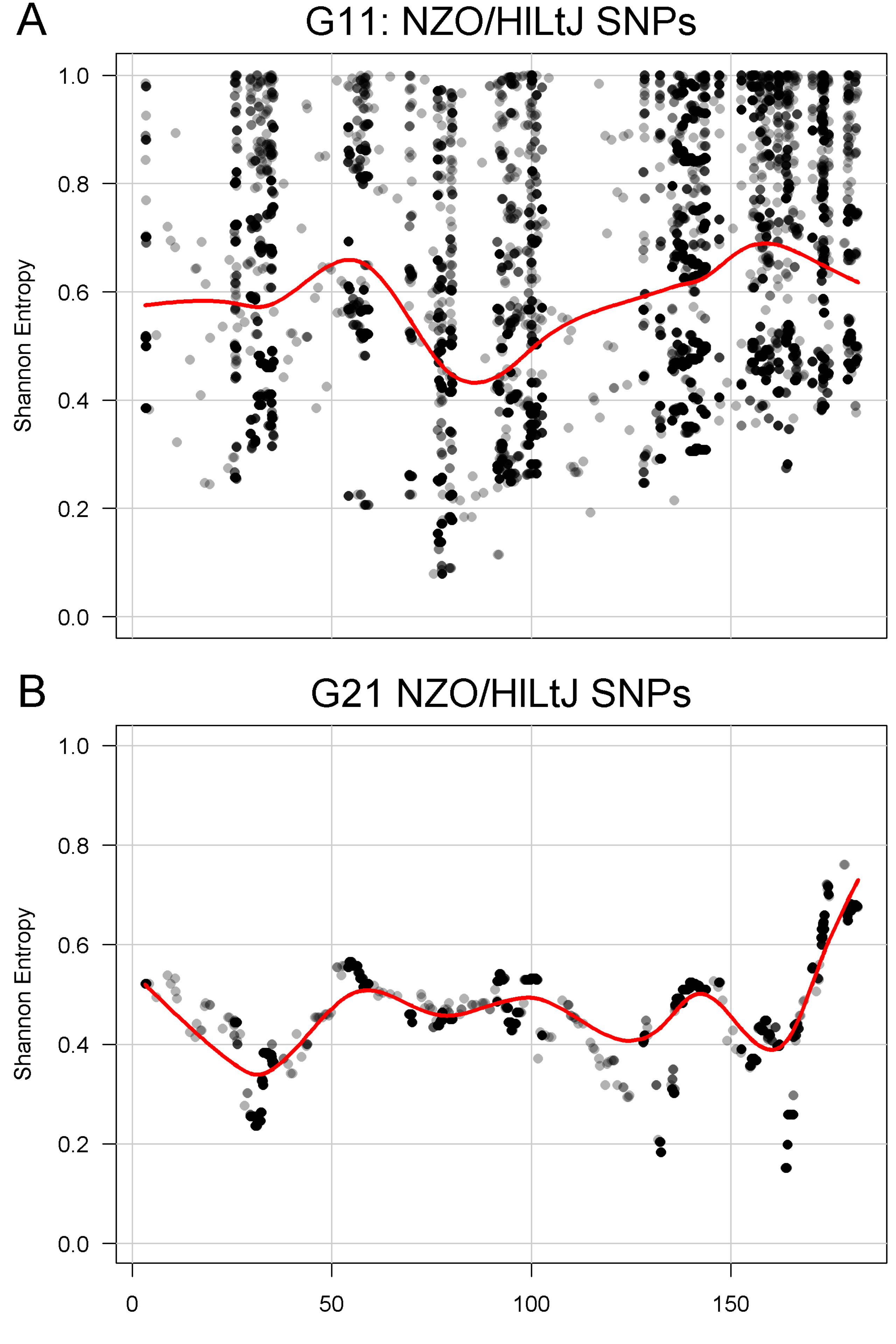
A
G11: NZO/HILtJ SNPs
Shannon Entropy
1.0
0.8
0.6
0.4
0.2
0.0
B
G21 NZO/HILtJ SNPs
Shannon Entropy
1.0
0.8
0.6
0.4
0.2
0.0
0
50
100
150

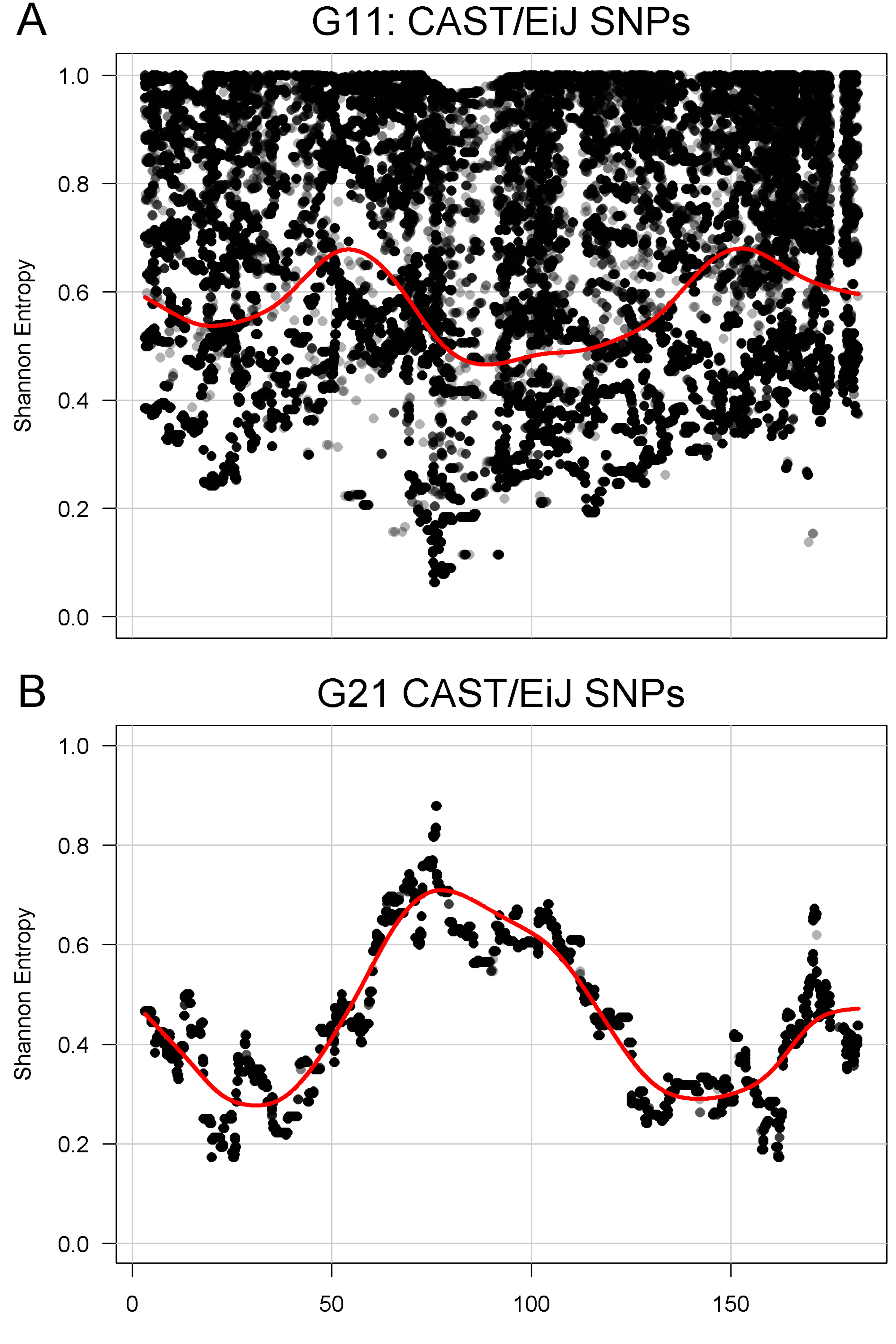
A
G11: CAST/EiJ SNPs
Shannon Entropy
1.0
0.8
0.6
0.4
0.2
0.0
B
G21 CAST/EiJ SNPs
Shannon Entropy
1.0
0.8
0.6
0.4
0.2
0.0
0
50
100
150

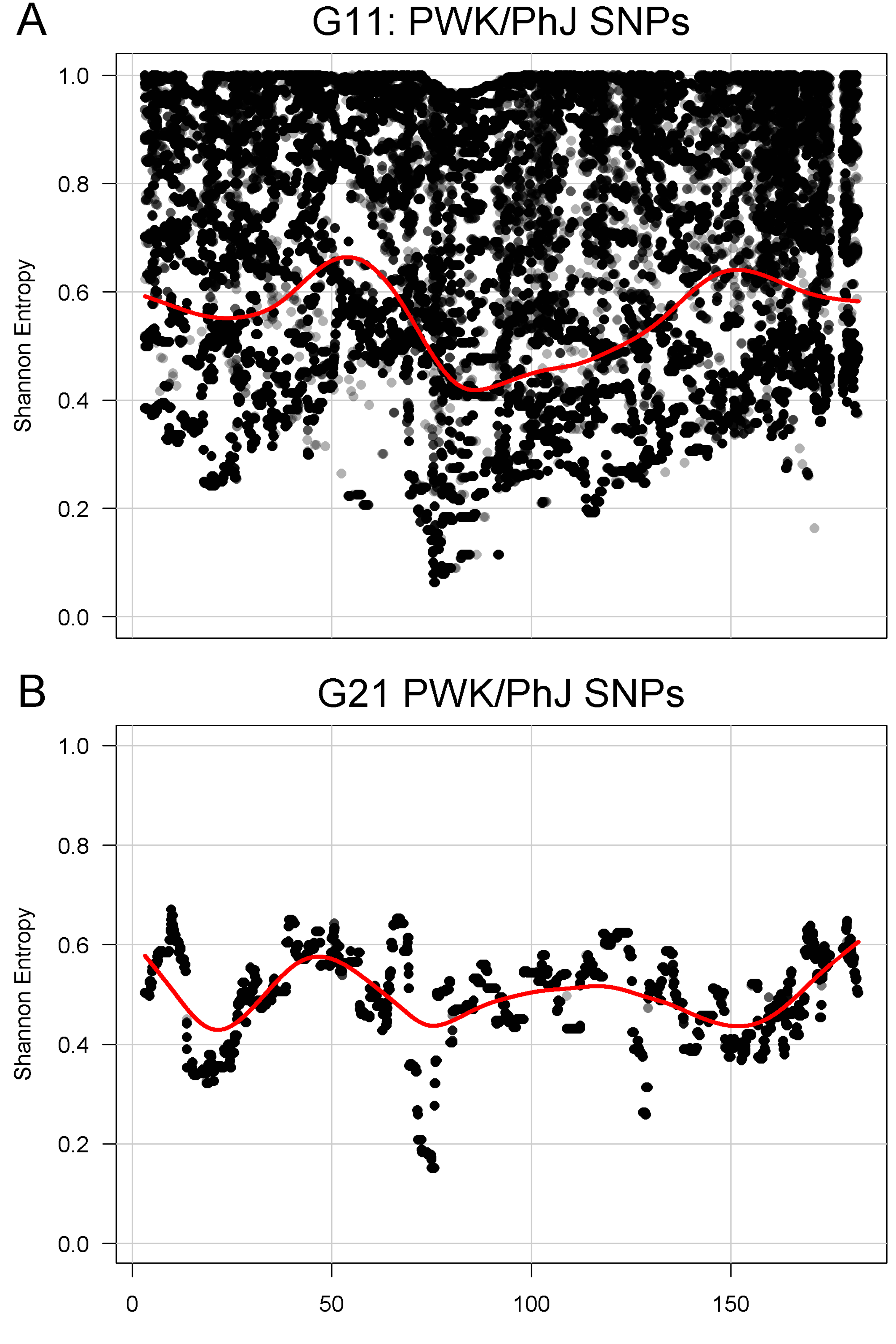
A
G11: PWK/PhJ SNPs
Shannon Entropy
1.0
0.8
0.6
0.4
0.2
0.0
B
G21 PWK/PhJ SNPs
Shannon Entropy
1.0
0.8
0.6
0.4
0.2
0.0
0
50
100
150

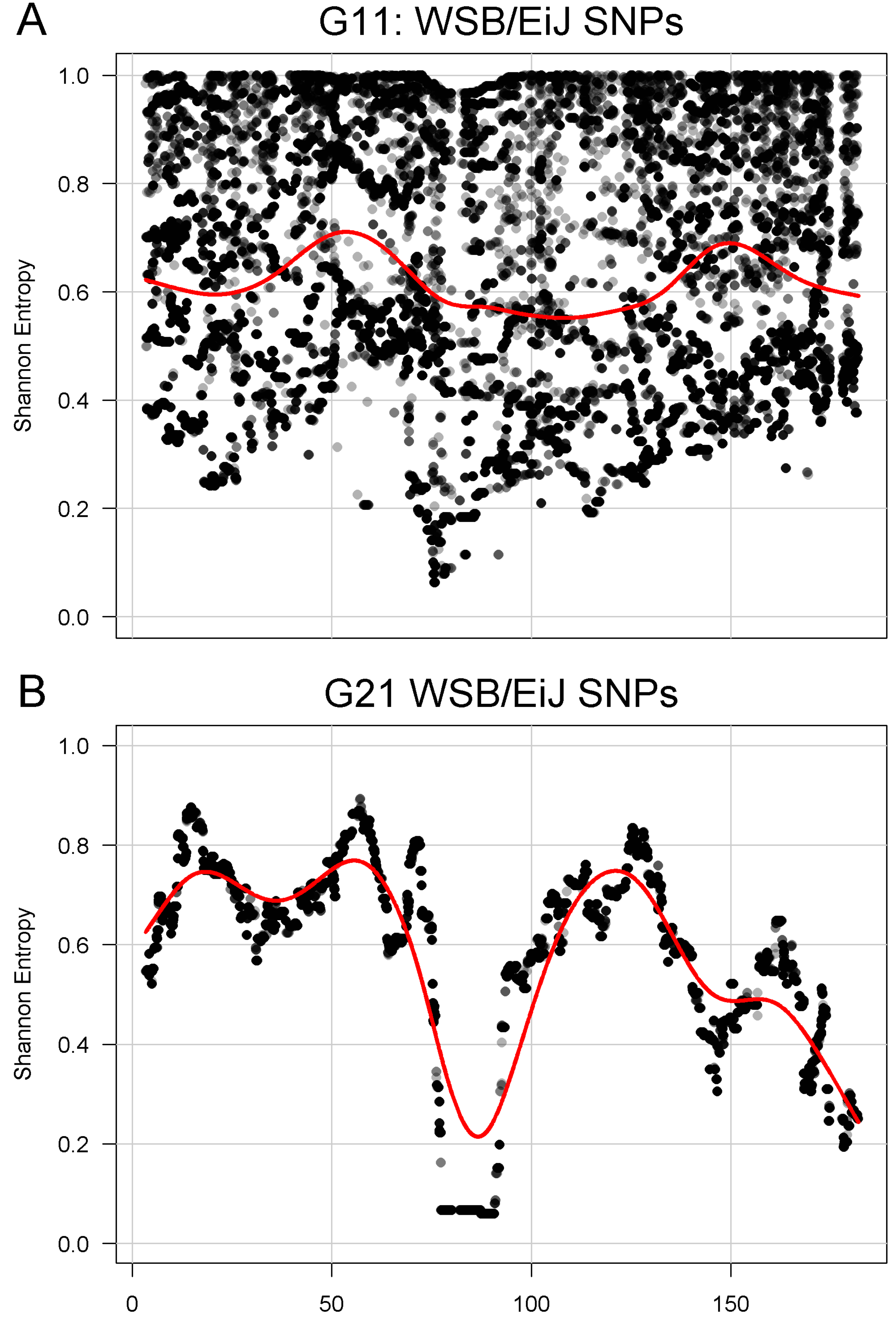
A
G11: WSB/EiJ SNPs
Shannon Entropy
1.0
0.8
0.6
0.4
0.2
0.0
B
G21 WSB/EiJ SNPs
Shannon Entropy
1.0
0.8
0.6
0.4
0.2
0.0
0
50
100
150
